# Celastrol Ameliorates Neuronal Mitochondrial Dysfunction Induced by Intracerebral Hemorrhage via Targeting cAMP‐Activated Exchange Protein‐1

**DOI:** 10.1002/advs.202307556

**Published:** 2024-03-14

**Authors:** Xiang Li, Wen Liu, Guannan Jiang, Jinrong Lian, Yi Zhong, Jialei Zhou, Haiying Li, Xingshun Xu, Yaobo Liu, Cong Cao, Jin Tao, Jian Cheng, John H Zhang, Gang Chen

**Affiliations:** ^1^ Department of Neurosurgery & Brain and Nerve Research Laboratory The First Affiliated Hospital of Soochow University 188 Shizi Street Suzhou 215006 China; ^2^ Institute of Stroke Research Soochow University 188 Shizi Street Suzhou 215006 China; ^3^ State Key Laboratory of Pharmaceutical Biotechnology School of Life Sciences Nanjing University 168 Xianlin Avenue Nanjing 210023 China; ^4^ Department of Neurology The First Affiliated Hospital of Soochow University 188 Shizi Street Suzhou 215006 China; ^5^ Jiangsu Key Laboratory of Neuropsychiatric Diseases and Institute of Neuroscience Soochow University Suzhou 215123 China; ^6^ Department of Physiology and Neurobiology Medical College of Soochow University Suzhou 215123 China; ^7^ Department of Physiology and Pharmacology School of Medicine Loma Linda University Loma Linda CA 92350 USA

**Keywords:** cAMP‐activated exchange protein‐1, celastrol, intracerebral hemorrhage, mitochondria, voltage‐dependent anion‐selective channel protein 1

## Abstract

Mitochondrial dysfunction contributes to the development of secondary brain injury (SBI) following intracerebral hemorrhage (ICH) and represents a promising therapeutic target. Celastrol, the primary active component of *Tripterygium wilfordii*, is a natural product that exhibits mitochondrial and neuronal protection in various cell types. This study aims to investigate the neuroprotective effects of celastrol against ICH‐induced SBI and explore its underlying mechanisms. Celastrol improves neurobehavioral and cognitive abilities in mice with autologous blood‐induced ICH, reduces neuronal death in vivo and in vitro, and promotes mitochondrial function recovery in neurons. Single‐cell nuclear sequencing reveals that the cyclic adenosine monophosphate (cAMP)/cAMP‐activated exchange protein‐1 (EPAC‐1) signaling pathways are impacted by celastrol. Celastrol binds to cNMP (a domain of EPAC‐1) to inhibit its interaction with voltage‐dependent anion‐selective channel protein 1 (VDAC1) and blocks the opening of mitochondrial permeability transition pores. After neuron‐specific knockout of EPAC1, the neuroprotective effects of celastrol are diminished. In summary, this study demonstrates that celastrol, through its interaction with EPAC‐1, ameliorates mitochondrial dysfunction in neurons, thus potentially improving SBI induced by ICH. These findings suggest that targeting EPAC‐1 with celastrol can be a promising therapeutic approach for treating ICH‐induced SBI.

## Graphical abstract

A mitochondrial protective compound, celastrol, is reported to exert neuroprotective effects following intracerebral hemorrhage (ICH). The results demonstrate that celastrol ameliorates neuronal mitochondrial dysfunction by interacting with EPAC‐1, potentially improving ICH‐induced secondary brain injury (SBI). These findings suggest that targeting EPAC‐1 with celastrol can be a promising therapeutic approach for the treatment of ICH‐induced SBI.

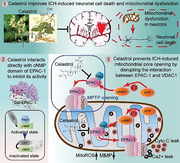

## 1 Introduction

Intracerebral hemorrhage (ICH), a type of hemorrhagic stroke resulting from bleeding within the brain tissue, is a significant contributor to morbidity and mortality globally.^[^ ^1^ ^]^ According to the 2019 Global Burden of Disease Study, ICH is responsible for ≈28% of all new strokes. The global incidence of ICH is estimated to be ≈42 per 100 000 individuals annually, with particularly high prevalence rates observed in certain regions of Oceania and Southeast Asia.^[^ ^2^ ^]^ Despite the improved surgical techniques leading to a decrease in mortality rates for patients with ICH, the occurrence of secondary brain injury (SBI) seriously impacts patient prognosis, with only ≈20% of patients regaining their ability to live independently after 6 months.^[^ ^3^ ^]^ This imposes a significant burden on both society and families. Currently, there is a lack of effective pharmacological interventions or preventative strategies for SBI following ICH in clinical practice. Therefore, the development of innovative therapeutic agents and targets to mitigate ICH‐induced SBI represents an urgent global medical challenge.

The mitochondria, acting as the “powerhouses” within nerve cells, play a pivotal role in maintaining cellular energy homeostasis in accordance with the unique energy requirements of the brain.^[^ ^4^ ^]^ In patients with ICH, the primary reduction in brain metabolism is responsible for decreased cerebral blood flow and hypoxic extraction around the hematoma, which leads to SBI. Hence, the involvement of mitochondria in SBI triggered by ICH is inevitable. In 2006, Prof. Michael N. Diringer proposed in an initial report that the occurrence of SBI in ICH patients is not attributed to ischemia or hypoxia but rather to mitochondrial damage, which has opened up a new avenue for ICH therapy.^[^ ^5^ ^]^ The neurons, serving as brain cells for stimulus perception and excitation transmission, exhibit high ATP consumption and are particularly vulnerable to mitochondrial damage. Thus, the preservation of mitochondria is crucial for neuronal survival. Previous studies have found that the structure and function of neuronal mitochondria remain relatively intact during the early stages of ICH. However, as ICH progresses, mitochondrial swelling, cristae fragmentation, the opening of the mitochondrial permeability transition pore (MPTP), and reduction in membrane potential occur.^[^ ^5^, ^6^ ^]^ Therefore, it is anticipated that early mitochondrial damage needs to be ameliorated and damaged neurons need to be repaired after ICH. Mitochondria thus represent a crucial drug target for SBI induced by ICH.

Celastrol, the primary active component of *Tripterygium wilfordii*, is a natural product that exhibits various biological activities including anti‐inflammatory, anti‐tumor, and anti‐obesity effects.^[^ ^4^, ^7^ ^]^ This compound demonstrates rapid in vivo absorption, prolonged half‐life, and high bioavailability. Furthermore, celastrol has been extensively studied for its ability to protect mitochondria. Specifically, celastrol inhibits the production of reactive oxygen species (ROS), improves oxidative stress, and reduces inflammation by safeguarding mitochondrial function.^[^ ^8^ ^]^ Moreover, celastrol induces mitophagy in macrophages via binding with Nur77 in the mitochondria to alleviate inflammation.^[^ ^9^ ^]^ Additionally, celastrol enhances the insulin resistance of hepatocytes through mitochondrial protection.^[^ ^10^ ^]^ Notably, celastrol has the ability to penetrate the blood–brain barrier and exert therapeutic effects in central nervous system diseases. Celastrol has been reported to possess antioxidative and anti‐inflammatory properties against cerebral ischemic‐reperfusion injury.^[^ ^7^, ^11^ ^]^ In Parkinson's disease, celastrol can alleviate motor deficits and nigrostriatal dopaminergic degeneration.^[^ ^12^ ^]^ Furthermore, celastrol ameliorates Alzheimer's disease by augmenting TFEB‐mediated autophagy and lysosomal biogenesis.^[^ ^13^ ^]^ Celastrol may thus serve as a promising mitochondrial protector in the context of central nervous system diseases. Celastrol is a compound with multiple cellular targets across various cell types. In addition to suppressing neuronal death, celastrol also exhibits inhibitory effects on ischemia‐induced pyroptosis in microglial cells and cell death in amyotrophic lateral sclerosis‐induced astrocytes. Furthermore, it demonstrates protective properties against endothelial cell death, thereby safeguarding the integrity of the blood–brain barrier.^[^ ^14^ ^]^ However, celastrol's potential to alleviate SBI induced by ICH remains unclear, and further investigation is necessary to clarify the underlying molecular mechanisms responsible for its protective effects on mitochondria.

In this study, we investigated the neuroprotective effects and underlying mechanisms of celastrol on SBI induced by ICH. Due to the high demand for ATP by neurons and their rich mitochondrial content in response to ischemia and hypoxia following ICH, our focus is specifically on neurons. Our data demonstrated that celastrol may serve as a promising mitochondrial protector to alleviate SBI caused by ICH, providing a potential therapeutic approach for ICH treatment. Moreover, we have identified a potential target for celastrol which could be an effective therapeutic strategy against ICH‐induced SBI.

## 2 Results

### 2.1 General Observations in Mice

No significant difference in body weight was observed between the sham group and the ICH group of mice. Furthermore, no statistical differences in the measurements of body temperature, and blood pressure between the sham group and the ICH group (data not shown). Representative coronal brain sections of mice from both sham and ICH (24 h) groups are shown in Figure S1A (Supporting Information).

### 2.2 Side Effects and Toxicity of Celastrol

**Figure 1:**
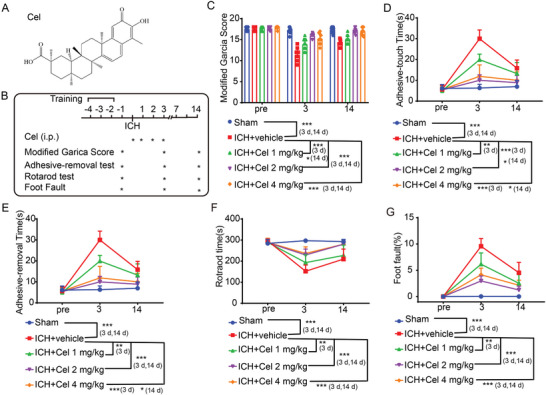
Celastrol ameliorated intracerebral hemorrhage (ICH)‐induced neurobehavioral deficits. A) The chemical structure of celastrol is depicted. B) A timeline illustrating drug administration and behavioral testing is presented. C) The modified Garcia Score test was used to assess the neurobehavioral impairment in mice. D,E) Adhesive removal test. The time taken by the mice to touch and remove the stickers was recorded and subjected to statistical analysis. F) Rotarod test. The latency to fall from a rotating drum after placement of the mice was measured. G) Foot fault test. The percentage of incorrect steps made by the left forelimb out of all steps taken by the mice was calculated. All data are presented as mean ± standard deviation (SD). Statistical significance was determined using two‐way analysis of variance (ANOVA) with Tukey's multiple comparisons test, ^*^ *p* < 0.05, ^**^ *p* < 0.001, ^***^ *p* < 0.0001, *n* = 10.

The chemical structure of celastrol, a pentacyclic triterpenoid compound, is depicted in **Figure** **1**A. As celastrol possesses anti‐tumor and apoptosis‐inducing properties, we investigated its potential toxicity in normal tissues and cells. We found that treatment with 10 µm of celastrol did not affect the activity of neurons, microglia, and astrocytes (Figure S1B, Supporting Information). Furthermore, mice were administered intraperitoneal injections of 5 mg k^−1^g celastrol once daily for 3 days. After 15 days, the heart, liver, spleen, lungs, and kidneys were subjected to hematoxylin and eosin (HE) staining to evaluate the celastrol toxicity. No long‐term alterations in cell morphology or organizational structure were observed in the major organs, thus demonstrating the favorable biosafety profile of celastrol (Figure S1C, Supporting Information). Furthermore, we extended our investigation to encompass blood routine examinations and liver function tests. Parameters, including red blood cells (RBC), white blood cells (WBC), hemoglobin concentration (HGC), platelet (PLT) count, as well as alanine transaminase (ALT) and aspartate transaminase (AST) levels, exhibited no significant differences compared to controls (Figure S1D,E, Supporting Information).

### 2.3 Celastrol Ameliorates ICH‐Induced Neurological Deficits in Mice

The effects of celastrol were examined in a mouse model of ICH. We assessed behavioral outcomes over the course of the ICH experiment. The timeline is presented in Figure 1B. First, the Modified Garcia score was recorded during neurological evaluations. The ICH group exhibited significant neurobehavioral impairment compared to the sham group, but this was partially alleviated by 2 and 4 mg k^−1^g celastrol treatment for 3 and 14 days (Figure 1C). Aside from the behavioral score, celastrol was also found to improve somatosensory and motor functions, as demonstrated by the positive results in adhesive‐removal, rotarod, and food fault detection tests. In the adhesive‐removal test, celastrol treatment significantly reduced the touch and removal times, indicating its potential to promote somatosensory and motor function recovery following ICH (Figure 1D,E). In addition, in the rotarod test, mice treated with celastrol remained on the accelerating beam for a longer duration compared to the vehicle‐treated mice following ICH, suggesting a partial restoration of motor function with celastrol (Figure 1F). The sensory function was evaluated using the foot fault test. The left forelimb exhibited a significant increase in the foot fault rate following ICH, indicating notable impairment of its sensory function. Treatment with 2 and 4 mg k^−1^g celastrol resulted in a reduction of the foot fault rate compared to the ICH group (Figure 1G). Overall, celastrol has the potential to ameliorate neurological deficits induced by ICH in a dose‐dependent manner, with an optimal intraperitoneal injection dosage of 2 mg k^−1^g for mice.

### 2.4 Celastrol Inhibits ICH‐Induced Neuronal Cell Death Both In Vivo and In vitro

**Figure 2:**
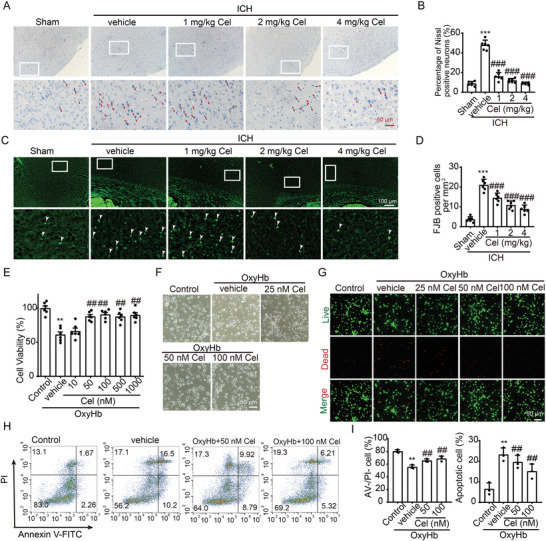
Celastrol attenuated ICH‐induced neuronal cell death. A) Nissl staining was used to assess neuronal morphology in the cortex, with arrows indicating aberrant Nissl bodies. B) The statistical analysis of abnormal Nissl bodies in the cortical region was conducted, *n* = 6. C) FJB staining is demonstrated, with arrows indicating the presence of FJB‐positive cells. D) The quantification of FJB‐positive cells in the perihematoma regions is presented, *n* = 6. The primary neurons were cultured and exposed to 10 µm oxyhemoglobin (OxyHb) for 12 h, followed by treatment with varying doses of celastrol for an additional 24 h. Subsequently, cells were harvested for the assessment of cell death indicators. E) Cell viability was assessed using the MTT assay, *n* = 6. F) The neuronal morphology was visualized under a microscope. G) Live‐dead cell staining was conducted to assess neuronal cell death. H) Annexin V and PI double staining combined with flow cytometry revealed neuronal apoptosis in various experimental groups in vitro. The presence of Annexin V without PI (PI^−^/Annexin V^+^) or the co‐presence of both Annexin V and PI (PI^+^/Annexin V^+^) indicated the occurrence of apoptotic neurons. I) The quantification of apoptotic cells was performed, *n* = 3. All data are presented as mean ± SD. Statistical significance was determined using one‐way ANOVA with Tukey's multiple comparisons tests (^*^ *p* < 0.05, ^**^ *p* < 0.001, ^***^ *p* < 0.0001 vs sham/control group; ^#^ *p* < 0.05, ^##^ *p* < 0.001, ^###^ *p* < 0.0001 vs vehicle group).

To further investigate the neuroprotective effects of celastrol on ICH‐induced SBI, Nissl, and Fluoro‐Jade B (FJB) staining were conducted in vivo. Neuronal Nissl bodies in the cortex were labeled by Nissl staining. In the sham group, the neurons in the cortex region exhibited a plump morphology with distinct dark blue Nissl bodies visible within their cytoplasm; conversely, in the ICH group, neuronal cells appeared crumpled and distorted with disrupted cell membranes, resulting in an absence of selective staining. With the administration of celastrol, the neurons in the cortical region were rescued in a manner that is dependent on the dosage (**Figure** **2**A,B). Also, celastrol treatment resulted in a significant reduction in FJB‐positive neurons induced by ICH, as demonstrated by the results of FJB staining at the perihematoma (Figure 2C,D).

Subsequently, primary neurons were isolated to assess the neuroprotective effects of celastrol. Upon stimulation with OxyHb, neuronal cell viability as detected by MTT assay was significantly reduced. Treatment with celastrol increased neuron activity in a dose‐dependent manner following OxyHb stimulation (Figure 2E). Additionally, the morphology of the neurons was observed, and the results were consistent with the MTT data (Figure 2F). Then, a live/dead cell viability assay was conducted to evaluate neuronal cell death in vitro, which revealed that treatment with 50 and 100 nm celastrol effectively attenuated OxyHb‐induced neuronal cell death (Figure 2G). Next, apoptotic neurons were labeled with Annexin V‐FITC and propidium iodide (PI) and subjected to flow cytometric analysis; the incidence of apoptotic cells increased following OxyHb treatment, whereas it decreased in response to 50 and 100 nm celastrol treatments (Figure 2H,I). In combination, celastrol exhibited the potential to counteract ICH‐induced neuronal cell death both in vivo and in vitro.

### 2.5 Celastrol Improved Mitochondrial Dysfunction Induced by ICH

**Figure 3:**
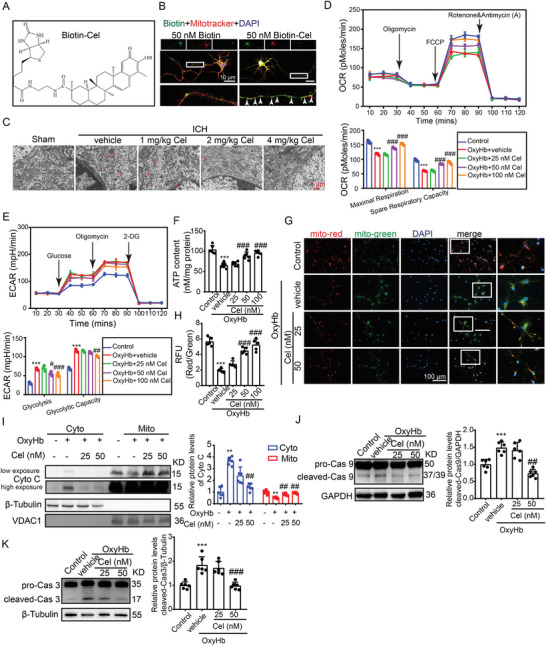
Celastrol improved neuronal mitochondrial dysfunction induced by ICH. A) The chemical structure of celastrol labeled with biotin is illustrated. B) Neurons were treated with 50 nm biotin or 50 nm Biotin‐Cel for 6 h, followed by immunofluorescence analysis using an anti‐biotin antibody (green) in neurons and mitotracker staining for mitochondria (red). Nuclei were stained with DAPI (blue). Representative images from triplicate experiments are shown. Arrows indicate the co‐localization of celastrol and mitochondria. Scale bar: 10 µm. C) Transmission electron microscopy of mitochondrial structures in neuronal cells of mice across different experimental groups. Scale bar: 5 µm. The neurons were stimulated with 10 µm OxyHb for 12 h and subsequently exposed to varying doses of celastrol treatment for 24 h. Afterward, the cells were collected for assessment of mitochondrial function. D) Oxygen consumption rates (OCR) were measured by using the Seahorse XF‐24 Extracellular Flux Analyze, *n* = 3. E) Extracellular acidification rate (ECAR) was measured by using the Seahorse XF‐24 Extracellular Flux Analyze, *n* = 3. F) The ATP content was measured using a chemiluminescence assay, *n* = 6. G) Neurons from various experimental groups were subjected to JC‐1 staining and subsequently visualized under a Nikon fluorescence microscope. Scale bar: 100 µm. H) Neurons from different experimental groups were subjected to JC‐1 staining, and the fluorescence intensity was quantified using a fluorescent microplate reader with excitation/emission wavelengths of 514/529 nm for monomers and 585/590 nm for aggregates, *n* = 6. I) Cytoplasm and mitochondria were separated to detect the protein levels of Cyto C, which was normalized to 1.0 based on the mean value of each protein in the control group. β‐Tubulin and voltage‐dependent anion‐selective channel protein 1 (VDAC1) served as loading controls. J) Neurons from various experimental groups were lysed, and western blot analysis was performed to determine the protein levels of caspase‐9. The mean value of each protein in the control group was normalized to 1.0, with GAPDH serving as the loading control, *n* = 6. K) Neurons from various experimental groups were lysed, and western blot analysis was performed to determine the protein levels of caspase‐3. The mean value of each protein in the control group was normalized to 1.0, with β‐Tubulin serving as the loading control, *n* = 6. All data are presented as mean ± SD. Statistical significance was determined using one‐ or two‐way ANOVA with Tukey's multiple comparisons tests (^*^ *p* < 0.05, ^**^ *p* < 0.001, ^***^ *p* < 0.0001 vs control group; ^#^ *p* < 0.05, ^##^ *p* < 0.001, ^###^ *p* < 0.0001 vs vehicle group).

Considering the mitochondrial protective effects of celastrol, its potential localization in mitochondria, and its ability to mitigate ICH‐induced mitochondrial damage were investigated. To investigate its mitochondrial localization, we utilized biotin‐labeled celastrol (Cel‐biotin, as depicted in **Figure** **3**A),^[^ ^15^ ^]^ revealing significant co‐localization with mitochondria (indicated by arrows), as opposed to the biotin‐only control group (Figure 3B). We wish to express our gratitude to Professor Yang Sun from Nanjing University for generously providing the biotin‐celastrol. In the ICH mouse model, brain tissue surrounding the hematoma was collected for electron microscopy. The results revealed that, following ICH, mitochondria exhibited swelling and deformation, while cristae junctions ruptured. However, treatment with celastrol demonstrated a significant rescue of mitochondrial structure and morphology compared to vehicle‐treated mice (Figure 3C). To reinforce the assertion that celastrol enhances mitochondrial integrity and function, we evaluated oxygen consumption rates (OCR) and extracellular acidification rates (ECAR). Consistently, exposure to OxyHb resulted in a substantial reduction in OCR compared to the control group. Importantly, treatment with 50 and 100 nm celastrol effectively reversed this OxyHb‐induced decline in OCR (Figure 3D). Additionally, celastrol demonstrated a significant reduction in OxyHb‐induced increase in ECAR in neurons (Figure 3E). To further evaluate the neuroprotective effect of celastrol on mitochondria, we measured the ATP content and found that 50 and 100 nm celastrol could mitigate OxyHb‐induced reduction in ATP levels (Figure 3F). Then, JC‐1 staining was employed to assess the mitochondrial membrane potential (MMP). Typically, JC‐1 aggregates exhibit red fluorescence in healthy mitochondria, whereas this signal is converted to green fluorescence in damaged mitochondria. As depicted in Figure 3G,H, a significant reduction in red fluorescence intensity and an elevation in green fluorescence intensity were detected in the neurons stimulation with OxyHb, as compared to the control group, indicating a decline in MMP and an opening of the MPTP. After celastrol treatment, the red fluorescence intensity was restored, while the green fluorescence intensity was attenuated compared to that of the OxyHb group. In addition to its role in ATP production, mitochondria also participate in the regulation of apoptosis. Therefore, we sought to investigate whether ICH could induce mitochondria‐mediated apoptosis in neurons. Our results revealed that OxyHb stimulation induced MPTP opening and subsequent release of cytochrome C into the cytoplasm, leading to cleavage of caspase 9, a key protein in the mitochondrial apoptotic pathway. These observations strongly suggest that OxyHb induces mitochondria‐mediated apoptosis. Remarkably, treatment with 50 nm celastrol significantly reversed these effects (Figure 3I,J). To further validate the execution of apoptosis, we assessed the presence of cleaved caspase‐3. Specifically, an increase in cleaved caspase‐3 levels was observed following OxyHb stimulation. Notably, celastrol treatment demonstrated the ability to inhibit this augmentation (Figure 3K).

Furthermore, one of the consequences of ICH is the deprivation of brain oxygen, leading to hypoxia. Therefore, we induced a hypoxic condition by treating it with cobalt chloride (10 µm for 48 h) and observed that neuronal mitochondria indeed suffered damage under hypoxia. Our findings demonstrate that celastrol can reverse the decline in MMP and ATP reduction under these hypoxic conditions (Figure S2, Supporting Information). These collective findings provide robust evidence supporting the notion that ICH‐induced mitochondrial dysfunction and neuronal apoptosis are effectively counteracted by celastrol treatment.

### 2.6 EPAC‐1 Was Identified as a Potential Target for Celastrol in Neurons by Single Nucleus RNA Sequence Analysis

**Figure 4:**
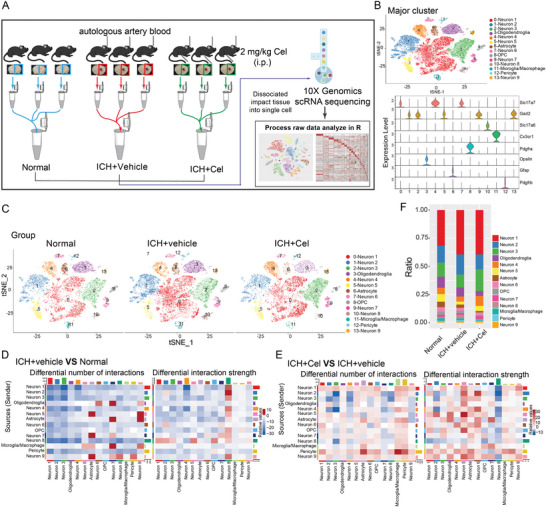
Single‐nucleus RNA sequencing was used to investigate the impact of celastrol on neuronal cells. After inducing ICH, mice were treated with 2 mg k^−1^g celastrol and brain tissue samples were collected 12 h post‐treatment. A) Schematic representation of single‐nucleus RNA sequencing. B) The *t*‐distributed stochastic neighbor embedding (*t*‐SNE) plot illustrates the major cell types in the mouse brain, while the violin plot shows the expression levels of specific markers within each cell type. C) The t‐SNE plot illustrates the clustering of single cells based on their cell types across different groups. D,E) The analysis of cell‐cell communication was conducted using CellChat, with a detailed heatmap displaying differential numbers and strengths of interactions. The bar plots colored on the top and right respectively represent the total values of columns (incoming signaling) and rows (outgoing signaling) displayed in the heatmap. The colorbar indicates that red (or blue) signifies an increase (or decrease) in signaling in the ICH+vehicle group compared to the Normal group(D); whereas it represents changes between ICH+Cel and ICH+vehicle groups (E). F) Variation in percentage change among different clusters within various groups.

To explore the potential target of celastrol, we performed single‐cell nucleus RNA sequencing (snRNA‐seq) (10× Genomics) on brain tissues from mice with a peripheral hematoma, as depicted in **Figures** **4**A and S3A (Supporting Information), which provided information on groupings, sampling sites, and layers. After undergoing quality control and filtering, a total of 44863 cells met the preprocessing threshold and were deemed suitable for downstream analysis (Figure S3B–E, Supporting Information). We performed principal component analysis (PCA) on genes with variable expression across all cells to investigate the cellular composition of brain tissues. This led to the identification of 14 main clusters, including neurons, oligodendroglia, astrocytes, microglia, microphages pericytes, and oligodendrocyte precursor cells. To identify each cell cluster, differential gene expression analysis was performed to generate cluster‐specific marker genes (Figure 4B,C; Figure S4, Supporting Information). The analysis of differential networks revealed a substantial reduction in both the number and strength of interactions following ICH compared to the Normal group (Figure 4D). Conversely, treatment with celastrol resulted in a marked increase in both interaction number and strength when compared to the ICH+vehicle group (Figure 4E). The site exhibited a predominance of neuron cells, with each group accounting for ≈75–85% of the total cell count (Figure 4F). As celastrol has demonstrated efficacy in mitigating ICH‐induced neuronal death, neurons would be selected for further analysis.

**Figure 5:**
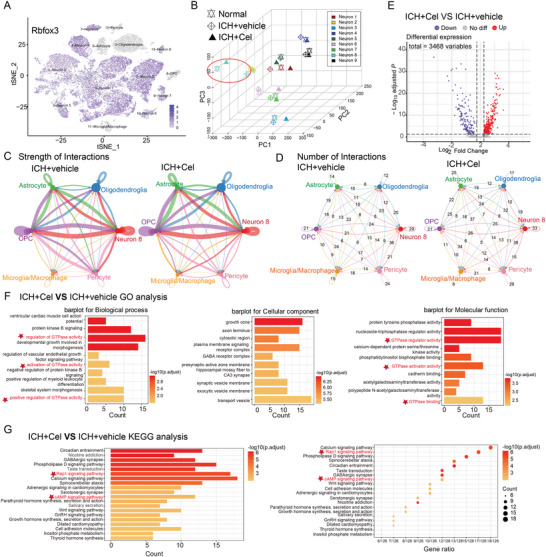
Enrichment analysis to identify potential target signaling pathway for celastrol on neurons. A) Rbfox3 was used as a marker for neurons. B) 3D‐ Principal component analysis (PCA) was employed to identify the subclusters with the highest variance. C) The strength of interactions between neuron 8 and other cell types was evaluated through CellChat analysis of intercellular communication. D) The analysis of cell–cell communication was performed using CellChat to demonstrate the quantification of interactions between neuron 8 and other cellular populations. E) A Gene Ontology (GO) enrichment analysis was performed to identify potential signaling pathways regulated by celastrol following ICH, with an average log_2_ fold change >0.25. F) Kyoto Encyclopedia of Genes and Genomes (KEGG) pathway analysis was conducted to identify potential signaling pathways regulated by celastrol following ICH, with an average log_2_ fold change >0.25.

Neurons were identified by the Rbfox3 marker, as depicted in **Figure** **5**A, and further classified into nine distinct subclusters. 3D PCA revealed distinct segregation between the transcriptional profiles of the ICH+cel group and ICH+vehicle groups. Notably, neuron 8 (cluster 10) cells exhibited the greatest separation along PC2, which accounted for the most variance in the data (Figure 5B). From Figure 4D,E, it is evident that the ICH+vehicle group exhibited a downregulation in the number of interactions between neuron 8 and other cells, while an upregulation was observed in interaction strength when compared to the Normal group. In contrast, the ICH+Cel group showed a slight upregulation in the number of interactions between neuron8 and other cells but a downregulation in interaction strength when compared to the ICH+vehicle group. In Figure 5C,D, neuron 8 was excluded from the analysis due to its communication with other cell types, yielding consistent results with those presented in Figure 4D,E. The results indicated that neuron 8 was indeed under the regulation of ICH and celastrol, which is consistent with Figure 5B. Therefore, neuron 8 was selected for differential gene expression analysis. A total of 4923 genes were found to be significantly upregulated or downregulated in the ICH+vehicle group compared with the Normal group, while a total of 3468 genes were found to be significantly upregulated or downregulated in the ICH+cel group compared with the ICH+vehicle group (Figure 5E; Figure S5A, Supporting Information). Then, Enriched terms and signaling pathways were identified using Gene Ontology (GO) and Kyoto Encyclopedia of Genes and Genomes (KEGG) pathway analysis. Enrichment analyses were conducted for both comparisons. GO analysis revealed enrichment of the GTPase regulator and activator activity functional pathway, while KEGG analysis showed significant enrichment of cAMP and Rap1 signaling pathway (ICH+vehicle vs Normal, Figure S5B,C). Similarly, GTPase activator and regulator activity functional pathways were also enriched, along with cAMP and Rap1 signaling pathways (ICH+Cel vs ICH+vehicle, Figure 5F,G). Thus, it can be deduced that the cAMP‐Rap1‐GTP signaling pathway is impaired in post‐ICH neurons, while celastrol has the ability to regulate this signaling cascade.

The crucial involvement of cAMP in signal‐metabolic coupling and mitochondrial ATP production is widely recognized. As a guanine nucleotide exchange factor specific to the Ras family of small molecule G proteins, cAMP‐activated exchange protein‐1 (EPAC‐1) activates Rap by replacing its inactive GDP form with active GTP, thereby enabling Rap to function as an important signaling molecule.^[^ ^16^ ^]^ Furthermore, EPAC‐1 possesses mitochondrial localization sequences that enable its direct involvement in the regulation of mitochondria.^[^ ^17^ ^]^ Therefore, EPAC‐1 is considered a potential regulatory target for celastrol in the context of ICH.

### 2.7 Celastrol Interacts Directly with the cNMP Domain of EPAC‐1

**Figure 6:**
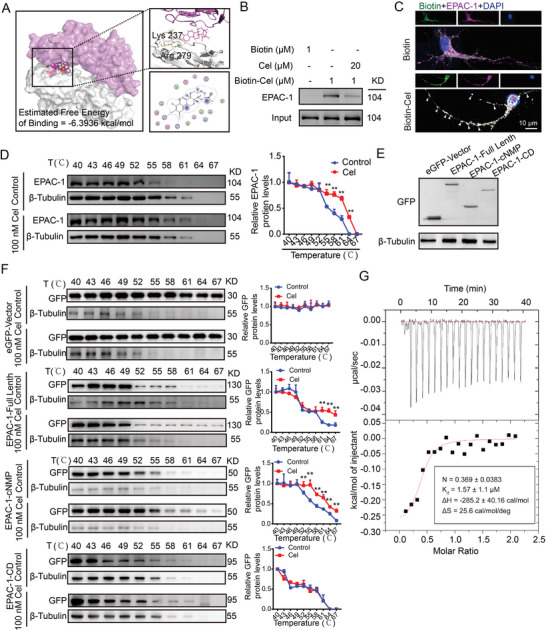
Celastrol interacts directly with the cNMP domain of EPAC‐1. A) A molecular docking analysis between celastrol and EPAC‐1 was conducted. B) Biotinylated‐protein interaction pull‐down assays were used to evaluate the interaction between celastrol and EPAC‐1. C) Immunofluorescence analysis was performed using an anti‐biotin antibody (green) and anti‐EPAC‐1 antibody (purple) to evaluate the co‐localization of celastrol and EPAC‐1. Nuclei were counterstained with DAPI (blue). Representative images from triplicate experiments are presented, with arrows indicating co‐localization. Scale bar: 10 µm. D) Primary neurons were subjected to celastrol treatment at a concentration of 50 nm for a duration of 6 h, followed by cellular thermal shift assay (CETSA) to evaluate the thermal stabilization of EPAC‐1 protein across varying temperatures. E) The 239T cells were transfected with an eGFP vector, full‐length EPAC‐1, EPAC‐1‐cNMP, and EPAC‐1‐CD plasmids. After transfection for 48 h, the cells were collected for western blot analysis to detect GFP expression. F) After transfection with different plasmids, the cells were subjected to treatment with or without 50 nm celastrol for 6 h. Subsequently, CETSA was performed to assess the thermal stabilization of GFP protein at varying temperatures. The mean value of each protein in the control group was normalized to 1.0, with β‐tubulin serving as the loading control. All data are presented as mean ± SD. Statistical significance was determined using one‐ or two‐way ANOVA with Tukey's multiple comparisons test (^*^ *p* < 0.05, ^**^ *p* < 0.001, ^***^ *p* < 0.0001 vs control group). G) The enthalpogram of the interaction between celastrol and cNMP‐EPAC‐1 at 25 °C was obtained using isothermal titration calorimetry (ITC). The titration curve was plotted as a function of the molar ratio between EPAC‐1 and the calculated concentration of celastrol in the assay.

First, molecular docking analysis revealed that celastrol probably binds to Lys 237 and Arg 279 of EPAC‐1, with an estimated free energy binding of −6.39 kcal mol^−1^ (**Figure** **6**A). Then, we performed biotin‐labeled celastrol (Cel‐biotin, Figure 3A) and biotinylated‐protein interaction pull‐down assays to demonstrate the binding of Cel‐biotin to EPAC‐1 in neuron cell lysates (Figure 6B). The fluorescence results were consistent with those obtained from the pull‐down assay (Figure 6C). Then, we confirmed the interaction between celastrol and EPAC‐1 through cellular thermal shift assay (CETSA). Our data demonstrated that celastrol significantly increased the thermal stability of EPAC‐1, even at elevated temperatures, compared to the control group (Figure 6D). To identify the binding domain of celastrol with EPAC‐1, we generated full‐length and truncated plasmids of EPAC‐1, including EPAC‐1 FL (2–881), cNMP (149–318), and CD (318–881). The plasmid design scheme and vector map are illustrated in Figure S6A,B (Supporting Information). The transfection efficiency and impact of the plasmid are presented in Figure S6C (Supporting Information) and Figure 6E. Following plasmid transfection, 293T cells were subjected to treatment with or without 100 nm celastrol, followed by CETSA analysis. The data revealed that cNMP (149–318) served as the binding domain for EPAC‐1 in the presence of celastrol, which is consistent with the predictions made in Figure 6A (Figure 6F). To further validate the binding domain, we expressed and purified the cNMP (149–318) protein (Figure S5D, Supporting Information). The results of isothermal titration calorimetry (ITC) analysis demonstrated that the dissociation constant (*K*_d_) value between celastrol and the cNMP domain of EPAC‐1 was 1.57 µm (Figure 6G). In conclusion, celastrol can indeed directly interact with the cNMP domain of EPAC‐1.

### 2.8 EPAC‐1 Participates in ICH‐Induced Mitochondrial Dysfunction by Interacting with Voltage‐Dependent Anion‐Selective Channel Protein 1(VDAC1)

**Figure 7:**
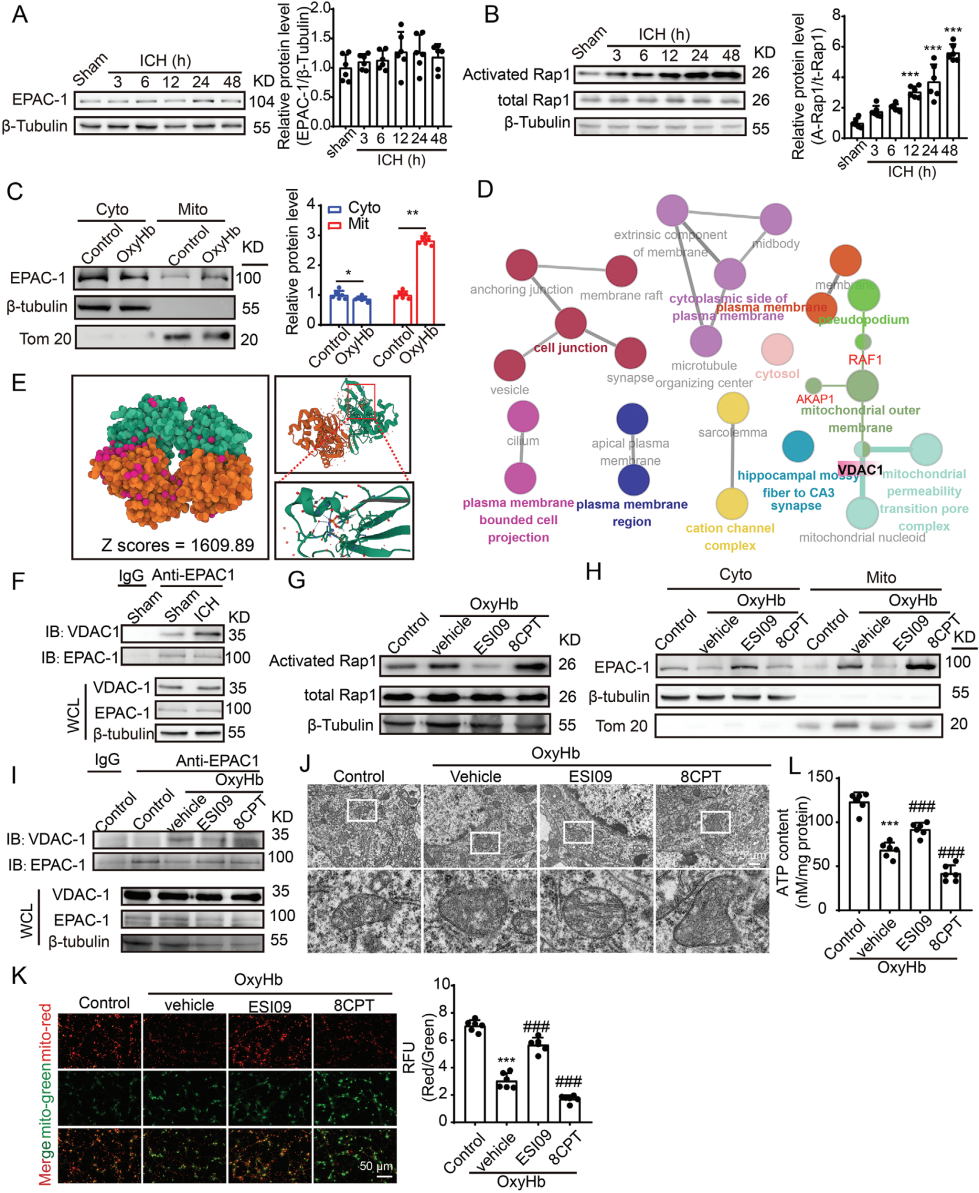
EPAC‐1 contributes to mitochondrial dysfunction induced by ICH through its interaction with VDAC1. A) Samples of cortex surrounding the hematoma in mice were collected at 3, 6, 12, 24, and 48 h post‐ICH induction. The protein levels of EPAC‐1 were assessed via western blot analysis, and the alterations in expression were quantified. β‐Tubulin served as loading controls, with a sham group being normalized to a value of 1.0 for accurate comparisons (*n* = 6). B) EPAC‐1 activation was assessed by measuring the levels of activated Rap1‐GTP using a Rap1 Activation Assay Kit. β‐Tubulin served as loading controls, with each sham group being normalized to a value of 1 for accurate comparisons *n* = 6. C) Neurons underwent a 12 h stimulation with 10 µm OxyHb. Subsequently, mitochondria and cytoplasm were isolated, and the protein levels of EPAC‐1 in both compartments were evaluated using western blot analysis. β‐Tubulin and Tom20 served as loading controls, with each control group being normalized to a value of 1 for accurate comparisons, *n* = 6. D) Protein‐protein interaction (PPI) network was queried from the STRING database (https://string‐db.org), followed by functional protein enrichment analysis conducted through Cytoscape and ClueGO. E) Rigid protein‐protein docking (ZDOCK) was conducted to investigate the relationship between EPAC‐1 and VDAC1. The PDB format of the protein structural domain was obtained from the Protein Data Bank (PDB) at http://www.rcsb.org/. The ZDOCK module was used to identify docking sites and calculate ZDOCK scores. F) Cortex samples surrounding the hematoma in mice were collected from sham and ICH 24 h groups, followed by co‐immunoprecipitation to validate the interaction between EPAC‐1 and VDAC‐1. G,H) Following stimulation with 10 µm OxyHb for 12 h, neurons were treated with the EPAC‐1 inhibitor ESI09 (10 µm) or activator 8CPT (10 µm) for 24 h. The levels of activated Rap1‐GTP were detected using a Rap1 Activation Assay Kit (G), while EPAC‐1 protein levels in both compartments were assessed via western blot analysis (H). Co‐immunoprecipitation was used to detect the interaction between EPAC‐1 and VDAC‐1 I), and mitochondrial structures were examined by means of transmission electron microscopy with a scale bar of 5 µm J). The ATP content was quantified L), and mitochondrial membrane potential (MMP) was measured by JC‐1 staining K). All data are presented as mean ± SD. Statistical significance was determined using one‐ or two‐way ANOVA with Tukey's multiple comparisons tests (*n* = 6, ^***^ *p* < 0.0001 vs control/ sham group; ^###^ *p* < 0.0001 vs Vehicle group).

To assess the impact of EPAC‐1 following ICH, we initially examined its protein levels post‐ICH and found no significant changes (**Figure** **7**A). Previous studies have suggested that cAMP can activate EPAC‐1 and subsequently activate Rap1.^[^ ^18^ ^]^ Therefore, we investigated the activation of Rap1 after ICH, which revealed an increase in its activity in the 12‐h period following ICH occurrence (Figure 7B). As reported, the translocation of EPAC‐1 is accompanied by the activation of Rap1 activity.^[^ ^18^ ^]^ Therefore, we observed the localization of EPAC‐1 and found that it translocated to mitochondria following OxyHb treatment (Figure 7C). To explore the involvement of EPAC‐1 in neuronal mitochondrial impairment following ICH, we employed protein–protein interaction (PPI) analysis to investigate the potential interacting proteins of EPAC‐1 (Figure S7, Supporting Information). Enrichment analysis of these proteins revealed that VDAC1, a protein associated with mitochondrial function and permeability, was implicated (Figure 7D). Subsequently, a rigid protein‐protein docking approach (ZDOCK) was employed to validate the interactions between EPAC‐1 and VDAC1. The PDB format for the protein structural domain was obtained from the Protein Data Bank (PDB) database (http://www.rcsb.org/). The ZDOCK module was used to identify the docking sites and compute the corresponding ZDOCK scores. As shown in Figure 7E, the ZDOCK score value was 1609.89, indicating that EPAC‐1 and VDAC1 formed a stable protein docking model. Additionally, the co‐precipitation of EPAC‐1 and VDAC was observed subsequent to ICH (Figure 7F). Based on the aforementioned findings, we hypothesized that EPAC1 may participate in post‐ICH neuronal mitochondrial impairment by activating Rap1 and modulating mitochondrial membrane permeability. To test this hypothesis, we employed EPAC‐1 inhibitors (ESI09) and activators (8CPT) to modulate Rap1 activity in neurons following OxyHb treatment (Figure 7G). Compared to the vehicle group, inhibition of EPAC‐1 activity resulted in decreased mitochondrial localization of EPAC‐1, while activation of EPAC‐1 led to increased mitochondrial localization (Figure 7H). The activation of EPAC‐1 also modulated the interaction between EPAC‐1 and VDAC1, as evidenced by the inhibition of EPAC‐1 activity and reduction in the formation of the EPAC‐1/VDAC1 complex. Conversely, upon activation, there was an increase in the association between EPAC‐1 and VDAC1 (Figure 7I). Moreover, inhibition of EPAC‐1 activity can mitigate neuronal mitochondrial swelling induced by OxyHb, characterized by irregular morphology, fractured cristae, and blurred structure, whereas activation exacerbates mitochondrial damage (Figure 7J). The same effects were observed on MMP and ATP production following treatment with ESI09 and 8CPT (Figure 7K,L). Consequently, EPAC‐1 is activated and subsequently interacts with VDAC‐1 to participate in the mitochondrial damage induced by ICH.

### 2.9 Celastrol Prevents Increased ICH‐Induced EPAC‐1 Activity and MPTP Opening

**Figure 8:**
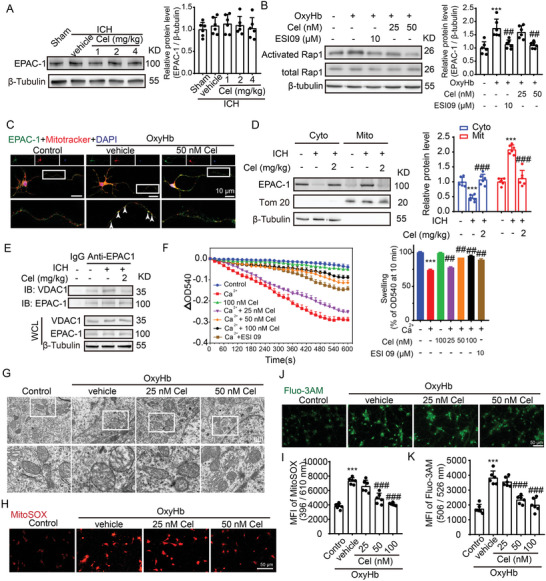
Celastrol inhibits the elevation of EPAC‐1 activity and MPTP opening induced by ICH. A) Samples of cortex surrounding the hematoma in mice were collected 48 h after ICH induction and treatment with 1, 2, and 4 mg k^−1^g celastrol. EPAC‐1 protein levels were detected by western blot. β‐Tubulin served as loading controls, with a sham group being normalized to a value of 1 for accurate comparisons, *n* = 6. B) After being stimulated with OxyHb, the neurons were treated with the EPAC‐1 inhibitor ESI09 (10 µm) or celastrol at concentrations of 25 and 50 nm. The activation levels of Rap1‐GTP were measured using a Rap1 Activation Assay Kit. β‐Tubulin served as loading controls, with each sham group being normalized to a value of 1 for accurate comparisons, *n* = 6. C) Following stimulation with OxyHb, the neurons were treated with 50 nm celastrol. Immunofluorescence analysis was performed using an anti‐EPAC‐1 antibody (green) to label neurons and mitotracker staining for mitochondria (red). Nuclei were counterstained with DAPI (blue). Representative images from triplicate experiments are presented, showing co‐localization of celastrol and mitochondria in the proximal neurite, as indicated by arrows. Scale bar: 10 µm. D) The protein levels of EPAC‐1 in the mitochondria and cytoplasm were evaluated by western blot analysis across different groups. The mean value of each protein in the control group was normalized to 1.0, with β‐tubulin and Tom 20 serving as the loading control, *n* = 6. E) Co‐immunoprecipitation was used to detect the interaction between EPAC‐1 and VDAC‐1. F) Mitochondria were pretreated with celastrol or ESI09 for 30 min, followed by exposure to Ca^2+^ for another 10 min. Mitochondria swelling traces were recorded based on the absorbance at 540 nm. The mitochondrial swelling was quantified, *n* = 6. The neurons were stimulated with 10 µm OxyHb for 12 h and subsequently exposed to varying doses of celastrol treatment for 24 h. Afterward, the cells were collected for assessment of mitochondrial function. G) Mitochondrial structures in neurons, with or without celastrol treatment following OxyHb stimulation, were observed using transmission electron microscopy. The scale bar was set at 5 µm. H) Neurons from various experimental groups were subjected to MitoSOX staining and subsequently visualized under a Nikon fluorescence microscope. I) Neurons from various experimental groups were subjected to MitoSOX staining, and the fluorescence intensity was quantified using a fluorescent microplate reader with excitation/emission wavelengths of 396/610 nm, *n* = 6. J) The Fluo3‐AM staining was used to detect calcium in the cytoplasm of neurons, which was subsequently visualized using a Nikon fluorescence microscope. K) The fluorescence intensity of Fluo3‐AM was measured using a fluorescent microplate reader with excitation and emission wavelengths set at 506 and 526 nm, respectively, *n* = 6. All data are presented as mean ± SD. Statistical significance was determined using one‐or two‐way ANOVA with Tukey's multiple comparisons tests (^*^ *p* < 0.05, ^**^ *p* < 0.001, ^***^ *p* < 0.0001 vs control group; ^#^ *p* < 0.05, ^##^ *p* < 0.001, ^###^ *p* < 0.0001 vs vehicle group).

Given that celastrol can directly interact with EPAC‐1, which in turn participates in ICH‐induced mitochondrial damage of neurons by activating rap1 and interacting with VDAC‐1, we investigated the regulatory effects of celastrol on EPAC‐1. Our data suggested that celastrol had no impact on the protein levels of EPAC‐1 (**Figure** **8**A). However, it significantly inhibits ICH‐induced enhancement of Rap 1 activity. The inhibition of Rap1 activity by 50 nm celastrol is comparable to that of 10 µm ESI09, an EPAC‐1 inhibitor (Figure 8B). Then, celastrol was found to reverse the mitochondrial localization of EPAC‐1 induced by ICH while also inhibiting the interaction between EPAC‐1 and VDAC‐1 caused by ICH (Figure 8C–E). Furthermore, celastrol exhibited a dose‐dependent ability to counteract Ca^2+^‐induced mitochondrial swelling in isolated mitochondria of mice brains, indicating its potential as a protective agent against Ca^2+^‐induced mitochondrial permeability transition (Figure 8F). This was further corroborated in the ICH model by electron microscopy, which demonstrated that OxyHb‐induced mitochondrial swelling and celastrol effectively reversed this phenomenon (Figure 8G). Due to mitochondrial damage and the opening of the MPTP, there is an increase in mitochondrial ROS and the release of calcium from mitochondria into the cytoplasm. Therefore, the levels of mitochondrial ROS and cytoplasmic calcium were quantified using MitoSOX Red and Fluo3‐AM fluorescent indicators, respectively. Stimulation with OxyHb resulted in a significant increase in mitochondrial ROS levels in neurons, which were effectively attenuated by celastrol treatment (Figure 8H,I). Similarly, treatment with 50 nm celastrol was found to effectively reverse the cytoplasmic Ca^2+^ overload induced by OxyHb (Figure 8J,K). The findings collectively suggest that celastrol exerts an inhibitory effect on EPAC‐1 activity, thereby mitigating the opening of MPTP triggered by ICH and ameliorating neuronal mitochondrial damage ensuing from this pathological condition.

### 2.10 Neuroprotective Effects of Celastrol Are Exerted Partly via EPAC‐1

**Figure 9:**
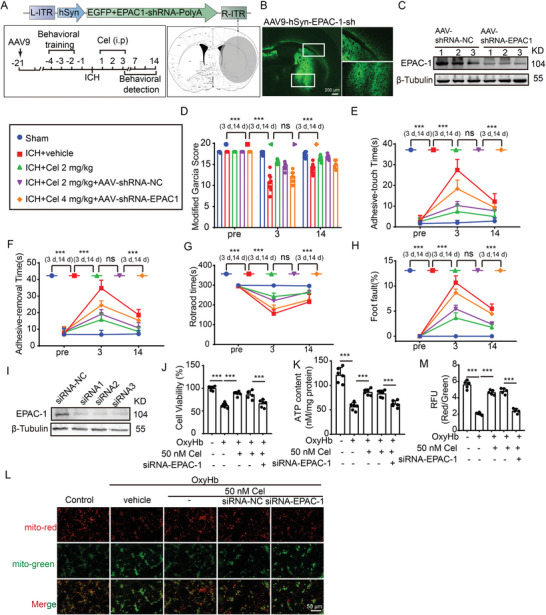
Celastrol‐induced neuroprotective effects partly depend on EPAC‐1. A) Schematic diagram depicting the experimental approach for EPAC‐1 knockdown. The efficacy of EPAC‐1 knockdown via AAV stereotactic injection was assessed by injecting AAV9‐EPAC‐1 shRNA‐eGFP (coordinates: 0.8 mm anterior to bregma, 2 mm left lateral, and 3.5 mm deep). The shaded region indicates the areas targeted by AAV intervention in this study, with details of the sampling procedure also provided. B) Representative micrographs depicting AAV9‐EPAC‐1 shRNA‐eGFP transfection in neurons are presented, showcasing characteristic features. The scale bar is 50 µm. C) western blot analysis was performed to compare the expression of EPAC‐1 between NC and shRNA groups. D) The modified Garcia Score test was used to assess the neurobehavioral impairment in mice. E,F) Adhesive removal test. The time taken by the mice to touch and remove the stickers was recorded and subjected to statistical analysis. G) Rotarod test. The latency to fall from a rotating drum after placement of the mice was measured. (H) Foot fault test. The percentage of incorrect steps made by the left forelimb out of all steps taken by the mice was calculated. All data are presented as mean ± SD. Statistical significance was determined using two‐way ANOVA with Tukey's multiple comparisons test, ^*^ *p* < 0.05, ^**^ *p* < 0.001, ^***^ *p* < 0.0001, *n* = 10. I) After transfection with siRNA‐EPAC‐1, primary cultured neurons were subjected to western blot analysis to compare the expression of EPAC‐1 between NC and siRNA groups. After transfection with siRNA‐EPAC‐1, primary cultured neurons were stimulated by 10 µm OxyHb for 12 h and treated with 50 nm celastrol for 24 h. J) The cell viability was assessed using the MTT assay. K) ATP content was detected. L) Neurons from various experimental groups were subjected to JC‐1 staining and subsequently visualized under a Nikon fluorescence microscope. M) Neurons from different experimental groups were subjected to JC‐1 staining, and the fluorescence intensity was quantified using a fluorescent microplate reader with excitation/emission wavelengths of 514/529 nm for monomers and 585/590 nm for aggregates. All data are presented as mean ± SD. Statistical significance was determined using one‐ or two‐way ANOVA with Tukey's multiple comparisons test (^*^ *p* < 0.05, ^**^ *p* < 0.001, ^***^ *p* < 0.0001 vs control group; ^#^ *p* < 0.05, ^##^ *p* < 0.001, ^###^ *p* < 0.0001 vs vehicle group; *n* = 6).

To further investigate the potential involvement of EPAC‐1 in the neuroprotective effects of celastrol, we conducted specific knockdown of EPAC‐1 in perihematoma and cortical neurons through stereotaxic injection of adeno‐associated virus (AAV) 9 with hSyn promoter (**Figure** **9**A). At 21 days post‐AAV administration, GFP signaling was observed in neurons surrounding the injection site (Figure 9B). In line with the fluorescence observation of GFP, a significant reduction in EPAC‐1 protein levels was observed 21 days after AAV injection (Figure 9C). Then, we evaluated the impact of celastrol on sensorimotor and motor functions in mice with EPAC‐1 knockdown using the Modified Garcia score (Figure 9D), adhesive removal test (Figure 9E,F), rotarod test (Figure 9G), and foot fault test (Figure 9H). The administration of the empty viral vector did not elicit any impact on the sensorimotor functions of ICH‐operated mice treated with celastrol, but EPAC‐1 knockdown significantly abrogated the neuroprotective effects of celastrol on ICH. Additionally, we employed siRNA to silence EPAC‐1 in primary cultured neurons (Figure 9I), demonstrating that the knockdown of EPAC‐1 did not impact neuronal cell viability or the MMP (Figure S8, Supporting Information). Consistent with the in vivo experiments, the knockdown of EPAC‐1 significantly reversed the protective effect of celastrol on neurons, as evidenced by reduced neuronal activity (Figure 9J), decreased ATP production (Figure 9K), and diminished MMP compared to the NC group (Figure 9L,M). These findings suggest that celastrol may alleviate neuronal damage caused by ICH, partly through its dependence on EPAC‐1, which is one of the targets of celastrol.

## 3 Discussion

ICH is a life‐threatening condition with high mortality and morbidity rates, resulting from the rupture of blood vessels within the brain parenchyma. Despite its devastating consequences, effective treatment options for ICH are limited, presenting significant challenges to clinicians and researchers.^[^ ^1^, ^19^ ^]^ Recently, significant advancements have been made in uncovering novel pathophysiological mechanisms underlying ICH, which may facilitate the development of therapeutic interventions and improve patient outcomes and quality of life.^[^ ^19^ ^]^ In this study, celastrol was found to exert neuroprotective effects against SBI induced by ICH. It has been reported that celastrol exhibits multifunctional properties in the treatment of various diseases. The therapeutic potential of celastrol has been extensively investigated in preclinical studies, demonstrating its efficacy in treating inflammatory diseases, autoimmune disorders, cancer, cardiovascular diseases, and neurological conditions.^[^ ^4^, ^12^, ^15^, ^20^ ^]^ Celastrol demonstrates neuroprotective effects in models of neurodegenerative and cerebrovascular diseases, including Alzheimer's disease,^[^ ^20e^ ^]^ Parkinson's disease,^[^ ^12^ ^]^ and ischemic stroke.^[^ ^7^, ^11^ ^]^ It exerts anti‐inflammatory and anti‐apoptotic effects while reducing oxidative stress and modulating the neuronal signaling pathways. These properties provide potential therapeutic benefits for neuronal injury. In addition, celastrol exerts some of its effects by modulating mitochondrial function. It can ameliorate cisplatin‐induced nephrotoxicity by improving mitochondrial function, activating the ROS‐mitochondrial fission‐mitophagy axis, promoting mitochondrial ubiquitination, activating an HSF‐PGC1a transcriptional axis, and participating in OPA1‐mediated mitochondrial fusion.^[^ ^7^, ^8^, ^9^, ^21^ ^]^ Consistent with this, our findings suggest that celastrol may exert a neuroprotective effect by preserving mitochondrial integrity in neurons, indicating its potential as a therapeutic agent for ICH‐induced SBI. However, celastrol exhibits a dual nature, with larger doses demonstrating anti‐tumor effects, while lower doses display anti‐inflammatory and antioxidant properties. In anti‐tumor investigations, celastrol (0.5–5 µm, in vitro) has been documented to induce ROS generation and calcium overload.^[^ ^4^, ^7^ ^]^ In our study, a comparatively low celastrol concentration (50 nm, in vitro) was employed. In the realm of neuroprotective and anti‐inflammatory research, our findings align with analogous results from existing literature, providing further affirmation of celastrol's consistent efficacy in mitigating excessive ROS generation and intracellular calcium overload.^[^ ^22^ ^]^ This body of evidence underscores the potential versatility of celastrol in modulating cellular responses, dependent on specific experimental conditions and concentrations. However, it is crucial to note that in any case, celastrol is intricately associated with mitochondria.

Since the publication of “Mitochondria make a comeback” in *Science* in 1999, there has been an increasing emphasis on mitochondrial research. Studies have demonstrated the significant role of mitochondria in ICH‐induced SBI, highlighting the need to investigate the molecular mechanisms underlying mitochondrial regulation following this condition.^[^ ^23^ ^]^ In this study, we used single‐cell sequencing to investigate the molecular mechanism underlying the mitochondrial protective effect of celastrol. Our findings suggest that celastrol may modulate the cAMP‐EPAC‐1 signaling pathway in response to ICH. cAMP, a widely distributed second messenger in cells, is capable of activating various factors to regulate mitochondrial functions. It has been reported that cAMP plays a crucial role in signal‐metabolic coupling and in the regulation of mitochondrial ATP production. Notably, the unique cAMP domain within the mitochondrial matrix is essential for modulating oxidative phosphorylation. Effector molecules of cAMP, including protein kinase A (PKA) and exchange proteins directly activated by cAMP (EPAC), exhibit diverse functions in different tissue mitochondria.^[^ ^16^, ^24^ ^]^ The functional and regulatory mechanisms of brain mitochondria through cAMP signaling may diverge from those observed in other tissue types. Despite this, there remains an insufficient understanding regarding the specific roles played by cAMP and its associated effectors with respect to modulating mitochondrial functionality within the nervous system. However, recent investigations suggest that EPAC‐1 exhibits superior efficacy compared to PKA in terms of regulating key aspects, such as MMP, ATP synthesis rates, and respiratory chain activity specifically within mouse cortical mitochondria.^[^ ^25^ ^]^ Our findings demonstrate that, following ICH, EPAC‐1 activity is upregulated, and it is translocated to the mitochondria, where it may interact with VDAC1 to modulate MPTP opening, resulting in mitochondrial dysfunction and neuronal death. In line with our findings, previous studies have demonstrated that EPAC‐1 is translocated to the mitochondria and regulates mitochondrial pore opening in cardiac ischemia‐reperfusion models. Mitochondria‐derived Ca^2+^ is then transferred to the endoplasmic reticulum via glucose‐regulated protein 75 (GRP75), leading to calcium ion overload and subsequent cardiomyocyte apoptosis.^[^ ^26^ ^]^ However, the mechanism by which EPAC‐1 regulates MPTP remains unclear. In our investigation, the PPI network indicated a potential interaction between EPAC‐1 and VDAC 1. This interaction was subsequently validated through Z‐DOCK, immunofluorescence, and immunoprecipitation experiments following ICH. However, further investigation is necessary to elucidate the binding sites of EPAC‐1 and VDAC1, and additional research into the role of EPAC‐1 in ICH pathogenesis is warranted. Targeting EPAC‐1 signaling may represent a promising therapeutic strategy for mitigating SBI following ICH, with the potential to identify novel small molecule agents that protect against neuronal mitochondrial damage.

EPAC‐1 is composed of five distinct regions, namely the Camp binding domain (cNMP) and the DEP domain (Dishevelled, Egl‐10, and Pleckstrin), which are used for cell membrane and mitochondria mapping, the REM domain (Ras‐association domain), the RA domain (Ras‐Association domain), and the CDC25 homologous sequence region (cAMP exchange activity).^[^ ^27^ ^]^ Its primary function is to serve as the specific guanine nucleotide exchange factor for the Ras family small molecule G protein Rap, facilitating the replacement of RAP‐bound GDP (inactive) with GTP to activate Rap and enable its crucial signaling role in cell survival, proliferation, and differentiation, among other processes.^[^ ^16^ ^]^ Our findings demonstrated that celastrol binds to the cNMP domain (cAMP binding area) of EPAC‐1, thereby regulating Rap1‐GTP activity and improving ICH‐induced SBI. The precise matching between the binding domain of EPAC‐1 and celastrol confirms their regulatory roles in neuronal Rap1‐GTP activity and mitochondrial function.

Significant efforts have been dedicated to enhancing the management of brain injury in SBI through preclinical investigations. However, owing to the intricate pathophysiology of ICH, safety concerns, and other factors, no neuroprotective agent has demonstrated efficacy in clinical trials. In this study, our findings demonstrate that celastrol exhibits promising therapeutic potential by ameliorating ICH‐induced mitochondrial damage and enhancing overall neuronal survival. However, the journey from the laboratory to clinical translation is a formidable challenge. There are still limitations in the clinical application of celastrol, such as its water solubility, biological half‐life, and dose range optimization. Therefore, it is imperative to employ high‐tech molecular modification and new formulation development. Also, the compound celastrol exhibits pleiotropic effects on various cell types, highlighting its potential as a multi‐target therapeutic agent. Our research primarily focuses on elucidating the neuroprotective mechanisms of celastrol in mitochondrial function, while future investigations will explore its protective effects and targets in other cell types, thereby addressing the broader implications of its therapeutic potential beyond neurons. Additionally, healthy adult male mice were utilized to establish the ICH model in this study; however, further investigations should include animals of different genders and ages. In addition, it is important to consider the potential impact of other relevant components in blood when simulating ICH in vivo, as only OxyHb was utilized in this study.^[^ ^28^ ^]^ Furthermore, additional investigation is required to explore the binding sites of EPAC‐1 and VDAC1. Moreover, it is necessary to examine the interaction between celastrol and EPAC‐1, as well as its potential impact on protein modification.

## 4 Conclusion

**Figure 10:**
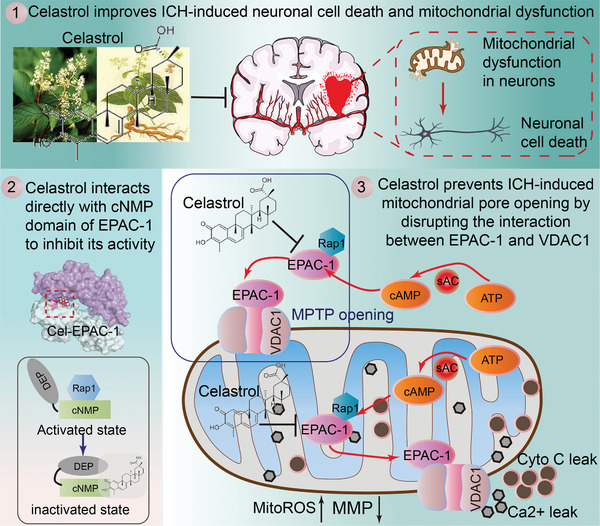
Graphic illustration of neuroprotective effects and mechanisms of celastrol. Following ICH, EPAC‐1 is activated within neurons and translocated to the outer membrane of mitochondria. Here, it can form a complex with VDAC1, promoting MPTP opening and subsequent collapse of mitochondrial membrane potential. This results in Ca^2+^ release, which induces neuronal apoptosis via cytochrome C (Cyto C). As a natural compound, celastrol can directly localize in mitochondria and interact with EPAC‐1 to modulate its activation, thereby impeding the binding of EPAC‐1 to VADC1. This ameliorates mitochondrial impairment in neuronal cells and exerts neuroprotective effects after ICH.

In conclusion, this study investigated the neuroprotective effects and mechanisms of celastrol on ICH‐induced SBI. Our findings suggest that celastrol may serve as a promising mitochondrial protector to alleviate SBI caused by ICH, providing a potential therapeutic approach for ICH treatment. Additionally, EPAC‐1 was found to be a target for celastrol, which can directly interact with VDAC1 and play a role in regulating mitochondrial function in neurons (**Figure** **10**). Therefore, EPAC‐1 represents a promising therapeutic target for the treatment of ICH.

## 5 Experimental Section

### Animals

The animal experiments were conducted in compliance with the recommendations of the Soochow University Laboratory Animal Center Animal Care and Use Guidelines. The study protocol was approved by the Ethics Committee of the First Affiliated Hospital of Soochow University (2018‐197). C57BL/6J male mice, aged 6–8 weeks and weighing 22–25 g, were obtained from the Experimental Animal Center of the Chinese Academy of Sciences (Shanghai, China).

### Reagents

Reagents used in this investigation are delineated in Table S1 (Supporting Information). Antibodies employed in this study are presented in Table S2 (Supporting Information).

### ICH Models

Mice were anesthetized with 3% (vol/vol) isoflurane using a gas anesthesia machine until they reached a surgical plane of anesthesia. Approximately 30 µL of blood was collected from the arteria caudalis. Then, the mice were maintained under nasal cone delivery of 1.5% v/v isoflurane anesthesia on a stereotaxic instrument. An ICH model was prepared by stereotactic injection of 30 µL of autologous blood into the right basal ganglia region.^[^ ^29^ ^]^ A small bone flap measuring 1 mm in diameter was drilled from the fontanelle, located 2.2 mm to the right and 0.1 mm anteriorly. Intraoperatively, the rectal temperature was maintained at a constant level of 37.0 ± 0.5 °C using a temperature‐controlled heating pad, and closure of the incision required 4‐0 silk sutures. The animals in the sham‐operated group underwent identical anesthesia and surgical procedures, followed by receiving 30 µL of phosphate buffer saline (PBS) injections.

### Drug Administration

In the experimental study, the mice were divided into three groups receiving celastrol at doses of 1, 2, and 4 mg k^−1^g. The corresponding concentrations were prepared by dissolving celastrol in DMSO (10 mg mL^−1^) and then diluting with PBS. Celastrol was administered intraperitoneally for 3 consecutive days after ICH according to different groups. The vehicle group received an intraperitoneal injection of PBS in equal volume.

### Neurobehavioral Tests

Neurobehavioral tests were conducted to assess neurological function using the Modified Garcia, adhesive touch and removal, rotarod, and foot fault test. The experimental animals underwent training three times a day for 3 days prior to surgery. Subsequently, experiments were repeated 1 day before surgery as well as on days 3 and 14 after ICH, with data being recorded.

### Neurobehavioral Tests—*Modified Garcia Test*

The Garcia scale for neurofunctional scoring was utilized to assess behavioral impairment, which included seven indicators: spontaneous activity, proprioception, tactile hair touch, limb symmetry, lateralization, forelimb extension, and climbing. These individual tests were scored on a scale from 0 to 3 by a blinded researcher.^[^ ^30^ ^]^

### Neurobehavioral Tests—*Adhesive Touch and Removal Test*

Tactile and sensorimotor responses were assessed via adhesive touch and removal experiments, in which 2 mm × 3 mm stickers were applied to the contralateral left front paw of mice with ICH. The time of initial contact with the sticker and the time of its removal from the affected paw were recorded to measure tactile response, with a maximum observation duration of 120 s.

### Neurobehavioral Tests—*Rotarod Test*

The mice were subjected to an accelerated rubber bar test, during which the speed of the bar increased from 5 revolutions per minute (rpm) to 40 rpm within a period of 300 s. Each mouse underwent three trials and the data were averaged.

### Neurobehavioral Tests—*Foot Fault Test*

The foot fault test was employed to identify forelimb placement dysfunction in mice, as previously described.^[^ ^31^ ^]^ The mice were positioned on an elevated metal grid surface (30 cm [L] × 35 cm [W] × 31 cm [H]) with a grid opening of 1.5 cm × 1.5 cm and recorded under the grid for 1 min. Each step taken by the subject was carefully monitored, as any misplacement or slippage of the left front paw between wires was considered a foot fault. A blinded investigator analyzed both the total number of steps and the frequency of foot defects occurring on the damaged limb's opposite side. The data were expressed as a percentage of foot defects to total steps for that specific claw.

### Nissl Staining

Nissl staining was utilized to evaluate the morphology of neuronal Nissl bodies. Following deparaffinization, the brain sections were subjected to Nissl staining at 60 °C for 30 min and subsequently fixed via reverse dehydration using a series of graded alcohols, followed by xylene treatment. Finally, the quantification of Nissl bodies in the perihematoma region was performed for each microscopic field.

### FJB Staining

FJB staining was employed to assess neuronal degeneration, as previously described.^[^ ^32^ ^]^ Brain sections were examined and imaged under a fluorescence microscope (Nikon, Japan) by an observer blinded to the experimental group. The FJB‐positive cells were analyzed using the ImageJ software.

### Primary Cortical Neuron Cultures

Primary neurons were isolated and cultured from 17‐day‐old rat embryos using the previously described method.^[^ ^33^ ^]^ The neurons were cultured with 50% of the medium replenished twice daily over a period of 1 week. Finally, the neurons underwent stimulation as specified before being collected for subsequent analysis.

### Primary Microglia and Astrocyte Cultures

Whole brains from 1‐day‐old rats were utilized for primary microglia and astrocyte extraction.^[^ ^32^ ^]^ Briefly, following the removal of meninges and blood vessels, the brain cortex underwent PBS washing and 0.25% trypsin digestion for 5 min at 37 °C. Trypsinization was halted using a complete DMEM/F12 medium. The brain tissue suspension was centrifuged, and cells were resuspended in a complete DMEM/F12 medium. Subsequently, cells were inoculated into 150 cm^2^ culture flasks with fresh medium, and half of the culture medium was renewed every 2 days. After an initial seeding period of 10–14 days, microglia were separated from astrocytes through shaking at 150 rpm for 4 h. Microglia settled down, while astrocytes remained suspended. Microglia and astrocytes were then subjected to indicated stimuli and collected for subsequent experiments.

### MTT Assay

Neurons were seeded in 96‐well plates at a density of 5 × 10^3^ cells per well and treated with varying concentrations of celastrol for 24 h. After washing with PBS, 5 mg mL^−1^ MTT solution was added and incubated for 4 h. The resulting formazan crystals were dissolved in DMSO, and the absorbance was measured at 560 nm using a Thermo Microplate reader.

### Live‐Dead Cell Staining

Neuronal cell death was assessed using a calcein‐AM/propidium iodide double‐staining kit (Thermo Fisher Scientific, Shanghai, China). Following oxyhemoglobin (OxyHb) stimulation, neurons were treated with celastrol or left untreated for 24 h. After being washed three times with PBS, the cells were incubated at 37 °C for 30 min in a prepared working solution (mixture of Calcein‐AM and PI) and observed under a fluorescence microscope (Nikon, Tokyo, Japan). In general, the green fluorescence of membrane‐permeable calcein AM indicates viable cells, while the red fluorescence of ethidium homodimer‐1 signifies dead cells.

### Annexin‐V and PI Staining

Neuronal apoptosis was assessed using the Apoptosis Detection Kit (Life Technologies, Gaithersburg, MD, USA) according to the manufacturer's instructions. Subsequently, flow cytometry analysis (Beckman, Miami, FL, USA) was performed on stained neurons with a minimum of 10 000 events recorded per sample.

### Immunofluorescence Assays

In vivo experiments involved the analysis of brain tissue sections embedded in paraffin, while in vitro experiments used paraformaldehyde fixation (4%) of neurons after specific incubation periods. The sections and cells were subsequently incubated with primary antibodies, while normal IgG was used as a negative control. Following this, suitable secondary antibodies were employed for immunostaining and visualization using either a fluorescence microscope (Nikon, Tokyo, Japan) or a laser scanning confocal microscope (ZEISS LSM 880, Carl Zeiss AG, Germany).

### Transmission Electron Microscope

The cortical tissues surrounding the hematoma were dissected into 1 mm^3^ fragments and fixed in a 2.5% glutaraldehyde solution for electron microscopy, as well as for neurons. The electron microscopy analysis was conducted by Sangon Biotech (Shanghai, China).

### Measurement of Mitochondrial Bioenergetics

The OCR and ECAR measurements were performed by Junli Biotechnology Technology Co., LTD (Shanghai, China). The XF24 Flux Analyzer (Seahorse Bioscience, North Billerica, Massachusetts, USA) was employed following the manufacturer's guidelines. The experimental protocol involved seeding 1×10^4^ primary neurons onto an XF96 well plate. Subsequent to seeding, sequential additions of oligomycin (1 µm), carbonyl cyanide‐4‐(trifluoromethoxy) phenylhydrazone (FCCP, 1 µm), rotenone (1 µm), and antimycin A (1 µm) were made to each well for OCR measurement in pmoles per minute. Additionally, ECAR levels in mpH per minute were assessed by the addition of 10 mm glucose, 2 µg oligomycin, and 5 mm 2‐DG. The experiments were conducted in triplicate for result validation.

### ATP Measurement

ATP content was evaluated using an ATP detection assay kit (Beyotime, Shanghai, China) in accordance with the manufacturer's instructions. Neurons were stimulated with or without OxyHb and subsequently treated with celastrol or left untreated for 24 h. Cells were collected and lysed using an ATP detection lysis buffer. The fluorescence intensity was quantified by a luminometer.

### MMP Detection

The MMP was evaluated through JC‐1 staining, utilizing a previously established protocol.^[^ ^34^ ^]^ Neurons were stimulated with OxyHb and treated with or without celastrol for 24 h. After being washed 3 times with PBS, the cells were incubated in a prepared JC‐1 working solution at 37 °C for 30 min. Subsequently, the stained cells were visualized under a fluorescent microscope (Tokyo, Japan), and fluorescence intensity was quantified using a fluorescent microplate reader (excitation/emission wavelengths of 514/529 nm for monomers and 585/590 nm for aggregates) (SpectraMax Paradigm, Thermo Fisher Scientific, USA).

### Mitochondria Isolation

Mitochondria were extracted using the Tissue and Cell Mitochondrial Extraction Kit (Beyotime, C3606, C3601) and separated from the cytoplasm through differential centrifugation of fresh brain tissue (processed within 1 h) and neuronal cells according to the manufacturer's instructions. The expression of the aforementioned proteins was subsequently detected by immunoblot analysis.

### Western Blot

Cell and tissue samples, collected from the vicinity of the hematoma, were lysed in lysis buffer (purchased from Beyotime Biotechnology Corp, Shanghai, China) supplemented with a cocktail of protease inhibitors. The quantification of total protein concentrations was performed using a BCA kit (Beyotime, Shanghai, China). Equal amounts of lysis product (20 µg protein/sample) were mixed with loading buffer and subjected to boiling at 100 °C for 10 min. The proteins were separated via SDS‐PAGE and subsequently transferred onto polyvinylidene difluoride membranes with a pore size of 0.45 µm (Millipore Corp., USA). The membranes were blocked with 5% BSA buffer for 1 h, followed by overnight incubation at 4 °C with the corresponding antibodies. After performing three rounds of PBST washing for 5 min each, the sample was incubated with the HRP‐labeled secondary antibody at room temperature for 1 h. The protein bands can be detected using the chemiluminescent reagents and semi‐quantified using ImageJ software.

### snRNA‐Seq Data Pre‐Processing and Quality Control

The snRNA‐seq data from each experiment were processed using Cell Ranger v5.0.0 pipeline (10× Genomics) based on the mouse reference genome GRCm38 (mm10).^[^ ^35^ ^]^ The digital gene expression matrices were subjected to analysis using the Seurat (v5.0.0) package in R (v4.2.4).^[^ ^36^ ^]^ Prior to downstream analysis, the cells underwent pre‐filtration based on UMI count (< 100000), number of genes (> 2500 and < 7500 genes), unique feature count (> 500), and percent.mt (< 10%).^[^ ^37^ ^]^ The normalization process involved SC Transform function with regression for percent.mt. For integration, it was identified 2000 highly variable genes that were shared using the Run harmony function.^[^ ^38^ ^]^ PCA and *t*‐Distributed Stochastic Neighbor Embedding were applied for dimensionality reduction, utilizing the top 50 principal components. Then proceeded by constructing a nearest‐neighbor graph based on the 30 dimensions obtained from PCA reduction using Find Neighbors, followed by clustering with Find Clusters at a resolution of 0.5.

### Cluster Marker Identification

The identification of cluster‐specific marker genes was performed using the Find All Markers function in the Seurat package on normalized gene expression data. Biological process enrichment analysis was conducted using DAVID (https://david.ncifcrf.gov/home.jsp) and Metascape (http://metascape.org) with the top 100 differentially expressed genes in each cluster or subset.

### Cell–Cell Communication Analysis

To infer and analyze cell–cell communication, CellChat (1.1.0) was utilized, a publicly available repository of ligands, receptors, cofactors, and their interactions.^[^ ^39^ ^]^ The versatile toolkit CellChat along with its web‐based Explorer (http://www.cellchat.org/) facilitate the construction of cell–cell communication atlases. For the analysis of cellular interactions, expression levels were standardized by normalizing to the total read count mapped to the same set of coding genes across all transcriptomes.

### Enrichment Analysis

Enrichment analysis was performed using the GO and KEGG databases to gain insights into the biological processes and pathways associated with the gene set of interest. The screening criteria included a nominal *p*‐value < 0.05, an FDR *q*‐value < 0.25, and avg_log2FC > 0.25. This allowed to identify overrepresented GO terms and KEGG pathways, providing a functional interpretation of the gene set.

### Molecular Docking

AutoDock (version 4.2.6) was used to perform molecular docking of celastrol and cAMP‐ EPAC‐1 proteins. To obtain the necessary information about proteins, the PDB format files were accessed from the RCSB Protein Data Bank. The SDF format file of celastrol was retrieved from the NCBI PubChem Compound database (https://www.ncbi.nlm.nih.gov/pccompound/). After solvent and ligand removal, as well as hydrogenation, electron transfer, and other operations, EPAC‐1 protein files were prepared for use as receptors. Then, a PDBQT format file of celastrol was established to serve as the ligand for subsequent molecular docking analysis. The results were analyzed using AutoDockTools (version 1.5.7), while PyMOL (version 2.4.1) was used for visual simulation.

### Pull‐Down of Celastrol‐Bind Proteins

Neuron lysates were incubated with biotin, celastrol‐biotin, or a combination of celastrol‐biotin and celastrol at 4 °C overnight. The lysates were subsequently subjected to pull‐down using streptavidin‐conjugated beads (Softlink soft release Avidin Resin, PROMEGA) at 4 °C for an additional 4 h. After extensively washing with PBS, the beads were boiled in 2 × loading buffer, and the supernatants were used for western blot analysis of EPAC‐1.

### CETSA

CETSA was conducted according to the previously reported methods.^[^ ^40^ ^]^ In brief, mice were administered 2 mg k^−1^g of celastrol for 1 day, while cells were incubated with celastrol for 6 h. Subsequently, brain tissues or cultured cells were collected and divided equally. The resulting samples underwent thermal denaturation at various temperatures using a PCR instrument, followed by three cycles of freeze‐thawing with liquid nitrogen and centrifugation at 20 000 g for 20 min at 4 °C. The supernatants obtained after centrifugation were transferred to new microtubes and subjected to western blot analysis.

### siRNA and Plasmid Construction

Full‐length and truncated EPAC‐1 plasmids were synthesized by Genescript Biotechnology (Nanjing, China), while EPAC‐1 siRNA constructs with the following sequences: siRNA1, 3′‐CCAGGCAGGAACCGATATA; siRNA2, 3′‐ GGGTCAGCGTACAGATGAA; and siRNA3, 3′‐GGTCAATTCTGCCGGTGAT were obtained from RiBobio (Guangzhou, China). The transfection of plasmids or siRNAs into HEK293T or neuron cells was carried out using Lipofectamine 3000 (Invitrogen, Carlsbad, CA, USA). The cells were subsequently incubated at 37 °C for 48 h, after which they were collected and prepared for subsequent experimentation.

### Purification of cNMP‐EPAC‐1

A segment of human EPAC‐1 (cNMP‐EPAC‐1) comprising amino acids 149–318 was cloned into a pGEX4T1 vector containing an N‐terminal GST‐tag that can be cleaved by thrombin. The resulting construct was transformed into Escherichia coli BL21 (DE3) cells and cultured overnight on Luria‐Bertani agar plates supplemented with ampicillin (100 µg mL^−1^). A single colony was isolated and inoculated into 10 mL of Luria‐Bertani medium supplemented with ampicillin. After incubation at 37 °C and 210 rpm for 6 h, the culture was scaled up to a volume of 2 L under identical conditions until the OD600 reached a value of 0.6. Subsequently, Isopropyl β‐D‐1‐thiogalactopyranoside (IPTG, 0.5 mm) was added to achieve a final concentration of 0.5 mm to induce protein expression, followed by further incubation at 18 °C for an additional period of 20 h. The supernatant was collected after harvesting and lysing the bacteria. The protein underwent purification through glutathione column followed by Superdex 75 gel filtration chromatography. Subsequently, HIS‐TEV protease cleavage was performed at a protein‐to‐protease ratio of 5:1 w/w for 16 h at 4 °C. The cleavage reaction mixture was subjected to sequential removal of GST‐tag and HIS‐TEV protease using glutathione column (GE, Healthcare, USA) and Ni‐NTA column (Qiagen, Duesseldorf, Germany) respectively. The protein was ultimately subjected to Superdex 75 gel filtration chromatography for further purification.

### ITC

The binding affinity between the cNMP‐EPAC‐1 protein and the compound was assessed through ITC using MicroCal iTC200 (GE Healthcare Life Sciences). The protein samples of cNMP‐EPAC‐1 were subjected to dialysis in a buffer solution containing 20 mm Tris‐HCl (pH 8.0), 100 mm NaCl, 0.1 mm Tris‐(carboxyethyl) phosphine hydrochloride (TCEP), and 0.5% DMSO. Celastrol was dissolved in the same buffer solution and injected into the calorimeter cell containing the cNMP‐EPAC‐1 solution (30 µm cNMP‐EPAC‐1 and 600 µm celastrol) with constant stirring at 200 g for titration involving aliquots of celastrol (1.0 µL). The independent fit model in NanoAnalyze was used to analyze the data, with uncertainties estimated using the native Statistics module through 1000 synthetic trials and a confidence level of 95%.

### Rap1 Activity Assay

Rap1‐GTP levels were quantified using the Rap1 Activation Assay Kit (NewEast Biosciences, 81 401) as described previously.^[^ ^41^ ^]^ Briefly, tissue and cell samples were incubated with an anti‐active Rap1 antibody and protein A/G agarose at 4 °C for 1 h. The agarose beads were precipitated by centrifugation at 5 000 g for 1 min, washed three times, and resuspended in 20 µL of 2 × loading buffer for 5 min before being collected for western blot detection.

### Co‐Immunoprecipitation Analysis

Protein samples were incubated overnight at 4 °C with agitation in the presence of either the indicated IgG (negative control) or corresponding antibodies (1 µg). The immune complex was subsequently precipitated using protein A/G agarose beads (Santa Cruz Biotechnology, Santa Cruz, CA, USA) and incubated for 4 h at 4 °C. The proteins that were immunoprecipitated were eluted using a loading buffer and subsequently analyzed via western blotting.

### Mitochondrial Swelling Detection

The test was conducted according to the methodology reported previously,^[^ ^42^ ^]^ with minor modifications. Brain mitochondria were isolated from each group of mice using commercially available mitochondrial isolation kits (Beyotime Biotechnology), and their concentrations were determined using BCA kits (Beyotime Biotechnology, Shanghai, China). Mitochondrial swelling was evaluated by measuring light scattering at 37 °C, utilizing a microplate reader (SpectraMax Plus, Molecular Devices, USA).

### Measurement of Mitochondrial Superoxide

Mito SOX Red (M36008, Invitrogen/Molecular Probes, USA) was used to evaluate the mitochondrial ROS production. After being washed three times with PBS, the cultured neurons were incubated with a working solution of 5 µm MitoSOX Red reagent at 37 °C for 10 min before being observed under a Nikon fluorescence microscope.

### Cytoplasmic Ca^2+^ Measurements

After stimulation, the neurons were incubated with a calcium‐ion fluorescent probe (Fluo‐3AM, Beyotime, China) at a concentration of 5 µm and maintained at 37 °C for 30 min. Subsequently, cell slides were washed thrice with PBS before being observed under a Nikon fluorescent microscope.

### Stereotaxic Injection of AAV

The neuron‐specific promoter hSyn was used to obtain the AAV9‐EPAC‐1 shRNA‐eGFP and the corresponding control AAV from Genechem. Stereotaxic injections were administered targeting the caudate nucleus, with the following coordinates: 0.8 mm anterior to bregma, 2 mm left lateral, and 3.5 mm deep. The mice were allowed a recovery period of 3 weeks after viral injection to ensure adequate transgene expression prior to experimentation.

### Statistics

All experiments were conducted in a randomized and blinded manner, with each experiment being independently repeated at least three times in vitro or with six animals per group in vivo. Statistical analysis was conducted using GraphPad Prism 8.0 software (San Diego, CA, USA), and the normality of the data was assessed using the D'Agostino‐Pearson's K2 test. The results were presented as mean ± standard deviation (SD). Parametric data analysis was performed using one‐way and two‐way analysis of variance (ANOVA), while differences in means across multiple groups over time were evaluated using two‐way repeated‐measures ANOVA. Statistical significance was set at *p* < 0.05.

## Conflict of interest

The authors declare no conflicts of interest.

## Supporting information

Supporting Information

## Data Availability

The data that support the findings of this study are available on request from the corresponding author. The data are not publicly available due to privacy or ethical restrictions.

## References

[1] a) J. Magid‐Bernstein , R. Girard , S. Polster , A. Srinath , S. Romanos , I. A. Awad , L. H. Sansing , Circ. Res. 2022, 130, 1204; 35420918 10.1161/CIRCRESAHA.121.319949 PMC10032582

[2] a) L. Puy , A. R. Parry‐Jones , E. C. Sandset , D. Dowlatshahi , W. Ziai , C. Cordonnier , Nat. Rev. Dis. Primers 2023, 9, 14; 36928219 10.1038/s41572-023-00424-7

[3] V. L. Feigin , C. M. Lawes , D. A. Bennett , S. L. Barker‐Collo , V. Parag , The Lancet. Neurology 2009, 8, 355. 19233729 10.1016/S1474-4422(09)70025-0

[4] a) X. Chen , Y. Zhao , W. Luo , S. Chen , F. Lin , X. Zhang , S. Fan , X. Shen , Y. Wang , G. Liang , Theranostics 2020, 10, 10290; 32929349 10.7150/thno.46728 PMC7481428

[5] J. S. Kim‐Han , S. J. Kopp , L. L. Dugan , M. N. Diringer , Stroke 2006, 37, 2457. 16960094 10.1161/01.STR.0000240674.99945.4e

[6] J. Qu , W. Chen , R. Hu , H. Feng , Oxid. Med. Cell. Longevity 2016, 2016, 2592935. 10.1155/2016/2592935 PMC4880716 27293511

[7] a) C. Y. Yan , S. H. Ouyang , X. Wang , Y. P. Wu , W. Y. Sun , W. J. Duan , L. Liang , X. Luo , H. Kurihara , Y. F. Li , R. R. He , Phytomedicine 2021, 80, 153398; 33130474 10.1016/j.phymed.2020.153398

[8] a) X. Yu , X. Meng , M. Xu , X. Zhang , Y. Zhang , G. Ding , S. Huang , A. Zhang , Z. Jia , EBioMedicine 2018, 36, 266; 30268831 10.1016/j.ebiom.2018.09.031 PMC6197337

[9] a) M. Hu , Q. Luo , G. Alitongbieke , S. Chong , C. Xu , L. Xie , X. Chen , D. Zhang , Y. Zhou , Z. Wang , X. Ye , L. Cai , F. Zhang , H. Chen , F. Jiang , H. Fang , S. Yang , J. Liu , M. T. Diaz‐Meco , Y. Su , H. Zhou , J. Moscat , X. Lin , X. K. Zhang , Mol. Cell 2017, 66, 141; 28388439 10.1016/j.molcel.2017.03.008 PMC5761061

[10] M. H. Abu Bakar , M. R. Sarmidi , J. S. Tan , M. N. Mohamad Rosdi , Eur. J. Pharmacol. 2017, 799, 73. 28161417 10.1016/j.ejphar.2017.01.043

[11] M. Jiang , X. Liu , D. Zhang , Y. Wang , X. Hu , F. Xu , M. Jin , F. Cao , L. Xu , J Neuroinflammation 2018, 15, 78. 29540209 10.1186/s12974-018-1124-6 PMC5853059

[12] C. Zhang , M. Zhao , B. Wang , Z. Su , B. Guo , L. Qin , W. Zhang , R. Zheng , Redox Biol. 2021, 47, 102134. 34600334 10.1016/j.redox.2021.102134 PMC8487081

[13] W. Zhang , J. Wang , C. Yang , Autophagy 2022, 18, 1740. 35253615 10.1080/15548627.2022.2046437 PMC9298436

[14] a) W. Dai , X. Wang , H. Teng , C. Li , B. Wang , J. Wang , Int. Immunopharmacol. 2019, 66, 215; 30472522 10.1016/j.intimp.2018.11.029

[15] Y. Zhu , N. Wan , X. Shan , G. Deng , Q. Xu , H. Ye , Y. Sun , Acta Pharm. Sin. B 2021, 11, 1200. 34094828 10.1016/j.apsb.2020.12.008 PMC8148064

[16] a) M. Gloerich , J. L. Bos , Annu. Rev. Pharmacol. Toxicol. 2010, 50, 355; 20055708 10.1146/annurev.pharmtox.010909.105714

[17] L. Pereira , H. Rehmann , D. H. Lao , J. R. Erickson , J. Bossuyt , J. Chen , D. M. Bers , Proc. Natl. Acad. Sci. USA 2015, 112, 3991. 25829540 10.1073/pnas.1416163112 PMC4386405

[18] P. Singhmar , X. Huo , N. Eijkelkamp , S. R. Berciano , F. Baameur , F. C. Mei , Y. Zhu , X. Cheng , D. Hawke , F. Mayor Jr. , C. Murga , C. J. Heijnen , A. Kavelaars , Proc. Natl. Acad. Sci. USA 2016, 113, 3036. 26929333 10.1073/pnas.1516036113 PMC4801297

[19] M. Zille , T. D. Farr , R. F. Keep , C. Romer , G. Xi , J. Boltze , EBioMedicine 2022, 76, 103880. 35158309 10.1016/j.ebiom.2022.103880 PMC8850756

[20] a) P. Luo , Q. Zhang , T. Y. Zhong , J. Y. Chen , J. Z. Zhang , Y. Tian , L. H. Zheng , F. Yang , L. Y. Dai , C. Zou , Z. J. Li , J. H. Liu , J. G. Wang , Mil Med Res 2022, 9, 22; 35596191 10.1186/s40779-022-00381-4 PMC9121578

[21] a) M. Liu , Y. Fan , D. Li , B. Han , Y. Meng , F. Chen , T. Liu , Z. Song , Y. Han , L. Huang , Y. Chang , P. Cao , A. Nakai , K. Tan , Mol. Oncol. 2021, 15, 2084; 33675143 10.1002/1878-0261.12936 PMC8334255

[22] a) T. L. Qing , L. Yan , S. K. Wang , X. Y. Dai , L. J. Ren , J. Q. Zhang , W. J. Shi , X. F. Zhang , M. T. Wang , J. K. Chen , J. B. Zhu , Ecotoxicol. Environ. Saf. 2023, 252, 114623; 36774793 10.1016/j.ecoenv.2023.114623

[23] a) Y. Zhang , S. Khan , Y. Liu , G. Wu , V. W. Yong , M. Xue , Front Immunol 2022, 13, 847246; 35355999 10.3389/fimmu.2022.847246 PMC8959663

[24] a) F. Valsecchi , L. S. Ramos‐Espiritu , J. Buck , L. R. Levin , G. Manfredi , Physiology 2013, 28, 199; 23636265 10.1152/physiol.00004.2013 PMC3870303

[25] E. Jakobsen , S. C. Lange , L. K. Bak , J. Neurosci. Res. 2019, 97, 1018. 31172581 10.1002/jnr.24477

[26] L. Fazal , M. Laudette , S. Paula‐Gomes , S. Pons , C. Conte , F. Tortosa , P. Sicard , Y. Sainte‐Marie , M. Bisserier , O. Lairez , A. Lucas , J. Roy , B. Ghaleh , J. Fauconnier , J. Mialet‐Perez , F. Lezoualc'h , Circ. Res. 2017, 120, 645. 28096195 10.1161/CIRCRESAHA.116.309859

[27] A. Kraemer , H. R. Rehmann , R. H. Cool , C. Theiss , J. de Rooij , J. L. Bos , A. Wittinghofer , J. Mol. Biol. 2001, 306, 1167. 11237625 10.1006/jmbi.2001.4444

[28] a) R. F. Keep , A. V. Andjelkovic , J. Xiang , S. M. Stamatovic , D. A. Antonetti , Y. Hua , G. Xi , J Cereb Blood Flow Metab 2018, 38, 1255; 29737222 10.1177/0271678X18774666 PMC6092767

[29] a) J. Jia , Z. Wang , M. Zhang , C. Huang , Y. Song , F. Xu , J. Zhang , J. Li , M. He , Y. Li , G. Ao , C. Hong , Y. Cao , Y. E. Chin , Z. C. Hua , J. Cheng , Sci. Adv. 2020, 6, eaaz5752; 32923620 10.1126/sciadv.aaz5752 PMC7449675

[30] X. C. Liu , L. Y. Jing , M. F. Yang , K. Wang , Y. Wang , X. Y. Fu , J. Fang , Y. J. Hou , J. Y. Sun , D. W. Li , Z. Y. Zhang , L. L. Mao , Y. M. Tang , X. T. Fu , C. D. Fan , X. Y. Yang , B. L. Sun , Cell. Mol. Neurobiol. 2016, 36, 647. 26224360 10.1007/s10571-015-0245-z PMC11482297

[31] J. Zhang , W. Zhang , X. Gao , Y. Zhao , D. Chen , N. Xu , H. Pu , R. A. Stetler , Y. Gao , J Cereb Blood Flow Metab 2019, 39, 1394. 29972653 10.1177/0271678X18785480 PMC6668518

[32] X. Li , T. Wang , D. Zhang , H. Li , H. Shen , X. Ding , G. Chen , Neuropharmacology 2018, 141, 305. 30218674 10.1016/j.neuropharm.2018.09.015

[33] C. H. Wu , C. C. Chen , T. H. Hung , Y. C. Chuang , M. Chao , S. K. Shyue , S. F. Chen , J Biomed Sci 2019, 26, 53. 31307481 10.1186/s12929-019-0543-8 PMC6628494

[34] L. Jin , Q. Cai , S. Wang , S. Wang , T. Mondal , J. Wang , Z. Quan , Cell Death Dis. 2018, 9, 1017. 30282996 10.1038/s41419-018-1064-1 PMC6170488

[35] A. Butler , P. Hoffman , P. Smibert , E. Papalexi , R. Satija , Nat. Biotechnol. 2018, 36, 411. 29608179 10.1038/nbt.4096 PMC6700744

[36] G. Yu , L. G. Wang , Y. Han , Q. Y. He , OMICS 2012, 16, 284. 22455463 10.1089/omi.2011.0118 PMC3339379

[37] T. Stuart , A. Butler , P. Hoffman , C. Hafemeister , E. Papalexi , W. M. Mauck 3rd , Y. Hao , M. Stoeckius , P. Smibert , R. Satija , Cell 2019, 177, 1888. 31178118 10.1016/j.cell.2019.05.031 PMC6687398

[38] M. Han , F. Li , Y. Zhang , P. Dai , J. He , Y. Li , Y. Zhu , J. Zheng , H. Huang , F. Bai , D. Gao , Cancer Cell 2022, 40, 1306. 36332622 10.1016/j.ccell.2022.10.011

[39] a) Z. Fang , Y. Tian , C. Sui , Y. Guo , X. Hu , Y. Lai , Z. Liao , J. Li , G. Feng , L. Jin , K. Qian , Front Cell Dev Biol 2022, 10, 919731; 35938159 10.3389/fcell.2022.919731 PMC9352955

[40] Y. Yang , X. Tan , J. Xu , T. Wang , T. Liang , X. Xu , C. Ma , Z. Xu , W. Wang , H. Li , H. Shen , X. Li , W. Dong , G. Chen , Biomed. Pharmacother. 2020, 126, 110044. 32114357 10.1016/j.biopha.2020.110044

[41] A. Krishnan , A. I. Bhasker , M. K. Singh , C. I. Rodriguez , E. C. Pérez , S. Altameemi , M. Lares , H. Khan , M. Ndiaye , N. Ahmad , S. M. Schieke , V. Setaluri , Mol. Cancer Res. 2022, 20, 1548. 35834616 10.1158/1541-7786.MCR-22-0026 PMC9532357

[42] J. Gao , J. Chen , X. Tang , L. Pan , F. Fang , L. Xu , X. Zhao , Q. Xu , J. Pharm. Pharmacol. 2006, 58, 227. 16451751 10.1211/jpp.58.2.0010

