# Correction to “Celastrol Ameliorates Neuronal Mitochondrial Dysfunction Induced by Intracerebral Hemorrhage via Targeting cAMP‐Activated Exchange Protein‐1”

**DOI:** 10.1002/advs.202406154

**Published:** 2024-07-10

**Authors:** Xiang Li, Wen Liu, Guannan Jiang, Jinrong Lian, Yi Zhong, Jialei Zhou, Haiying Li, Xingshun Xu, Yaobo Liu, Cong Cao, Jin Tao, Jian Cheng, John H. Zhang, Gang Chen

*Adv. Sci*. **2024**, e2307556.

https://doi.org/10.1002/advs.202307556

Description of errors:

1. The original Figure 1 contain an error in Figure 1E. The revised Figure 1E is shown below.

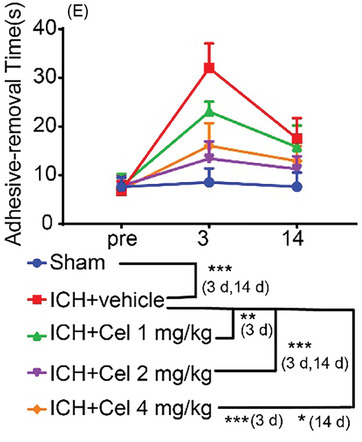

2. The figure legends of Figures 1, 2, 3, 6, 7, 8, and 9 contained errors in the description of statistical significance. The incorrect descriptions “** *p* < 0.001, *** *p* < 0.0001” and “## *p* < 0.001, ### *p* < 0.0001” has been corrected to “** *p* < 0.01, *** *p* < 0.001” and “## *p* < 0.01, ### *p* < 0.001”.
3. In the original figure, the *x*‐axis of Figure 5E was erroneously read. The *x*‐axis has now been correctly changed. Additionally, the figure legend of Figure 5 ha missing items and order problems. The revised figure legend for Figure 5 is shown below.

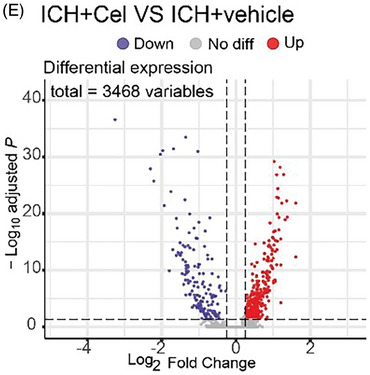


Figure 5 Enrichment analysis to identify potential target signaling pathway for celastrol on neurons. A) Rbfox3 was used as a marker for neurons. B) 3D‐Principal component analysis (PCA) was employed to identify the subclusters with the highest variance. C) The strength of interactions between neuron 8 and other cell types was evaluated through CellChat analysis of intercellular communication. D) The analysis of cell–cell communication was performed using CellChat to demonstrate the quantification of interactions between neuron 8 and other cellular populations. E) The volcano plot illustrates the differential expression of genes between the ICH+Cel group and the ICH+vehicle group. F) A Gene Ontology (GO) enrichment analysis was performed to identify potential signaling pathways regulated by celastrol following ICH, with an average log2 fold change >0.25. G) Kyoto Encyclopedia of Genes and Genomes (KEGG) pathway analysis was conducted to identify potential signaling pathways regulated by celastrol following ICH, with an average log2 fold change >0.25.

These changes do not affect the results or conclusions of this study. The authors apologize for any inconvenience caused.

