# Supplementary material for: Celastrol Ameliorates Neuronal Mitochondrial Dysfunction Induced by Intracerebral Hemorrhage via Targeting cAMP‐Activated Exchange Protein‐1
Source: Adv Sci (Weinh). 2024 Mar 14;11(19):2307556. doi: 10.1002/advs.202307556 (PMC11109624; doi:10.1002/advs.202307556)
Supplement: Supplementary file 1 — Supporting Information [file ADVS-11-2307556-s001.pdf]

ADVANCED SCIENCE
Open Access

Supporting Information

for *Adv. Sci.*, DOI 10.1002/advs.202307556

Celastrol Ameliorates Neuronal Mitochondrial Dysfunction Induced by Intracerebral Hemorrhage via Targeting cAMP-Activated Exchange Protein-1

Xiang Li Sr., Wen Liu, Guannan Jiang, Jinrong Lian, Yi Zhong, Jialei Zhou, Haiying Li, Xingshun Xu, Yaobo Liu, Cong Cao, Jin Tao, Jian Cheng, John H Zhang and Gang Chen**

Supporting Information

Celastrol Ameliorates Neuronal Mitochondrial Dysfunction Induced By Intracerebral Hemorrhage Via Targeting cAMP-activated Exchange Protein-1

Xiang Li, Sr. [#,*,1,2], Wen Liu [#,3], Guannan Jiang[#,1,2], Jinrong Lian[1,2], Yi Zhong[1,2], Jialei Zhou[1,2], Haiying Li[1,2], Xingshun Xu[4,5], Yaobo Liu[5], Cong Cao[5], Jin Tao[5,6], Jian Cheng[5], John H Zhang[7], Gang Chen[*,1,2]

[1]Department of Neurosurgery & Brain and Nerve Research Laboratory, The First Affiliated Hospital of Soochow University, 188 Shizi Street, Suzhou 215006, China.
[2]Institute of Stroke Research, Soochow University, 188 Shizi Street, Suzhou 215006, China.
[3]State Key Laboratory of Pharmaceutical Biotechnology, School of Life Sciences, Nanjing University, 168 Xianlin Avenue, Nanjing 210023, China
[4]Department of Neurology, The First Affiliated Hospital of Soochow University, 188 Shizi Street, Suzhou 215006, China.
[5]Jiangsu Key Laboratory of Neuropsychiatric Diseases and Institute of Neuroscience, Soochow University, Suzhou 215123, China.
[6]Department of Physiology and Neurobiology, Medical College of Soochow University, Suzhou 215123, China.
[7]Department of Physiology and Pharmacology, Loma Linda University, School of Medicine, CA
[#]These authors contributed equally to this work.

Table S1 Antibody information

Antibody	Full name	Specificity	Citation	Type	Host	Dilution	Company	City	Country
VDAC1	Anti-VDAC1/Porin Antibody	Mouse, Rat, Human	ab186321	monoclonal antibody	Mouse	1:2000	Abcam	Cambridge	UK
Caspase-9	Anti-Caspase-9 Antibody	Mouse, Rat	ab184786	monoclonal antibody	Rabbit	1:2000	Abcam	Cambridge	UK
Cytochrome C	Anti-Cytochrome C Antibody	Mouse, Rat, Human	ab133504	monoclonal antibody	Rabbit	1:2000	Abcam	Cambridge	UK
Epac1	Anti-Epac1 Antibody	Mouse, Rat, Human	ab109415	monoclonal antibody	Rabbit	1:2000	Abcam	Cambridge	UK
NeuN	Anti-NeuN Antibody	Mouse, Rat, Human	ab177487	monoclonal antibody	Rabbit	1:2000	Abcam	Cambridge	UK
Epac1	Anti-Epac1 Antibody	Human	ab21236	polyclonal antibody	Rabbit	1:2000	Abcam	Cambridge	UK
Tom20	Tom20 Antibody	Mouse, Rat, Human	42406s	monoclonal antibody	Rabbit	1:2000	Cell Signaling Technology	Boston	USA
EPAC1	Anti-Epac1 Antibody	Mouse, Rat, Human	4155s	monoclonal antibody	Mouse	1:2000	Cell Signaling Technology	Boston	USA
Rabbit IgG	Anti-rabbit IgG Antibody	Mouse, Rat, Human	7074s	polyclonal antibody	Rabbit	1:2000	Cell Signaling Technology	Boston	USA
Mouse IgG	Anti-mouse IgG Antibody	Mouse, Rat, Human	7076s	polyclonal antibody	Mouse	1:2000	Cell Signaling Technology	Boston	USA
Tubulin	Tubulin beta Antibody	Mouse, Rat, Human	AF7011	polyclonal antibody	Rabbit	1:2000	Yeasen Biotechnology	Shanghai	China
GFP	Anti-GFP/eGFP-tag Antibody	Mouse, Rat, Human	AP52804	monoclonal antibody	Mouse	1:1000	Abcepta	Suzhou	China
EPAC1	Anti-Epac1 Antibody	Mouse, Rat, Human	sc-28366	monoclonal antibody	Mouse	200 µg/ml	Santa Cruz Biotechnology	Dallas	USA
Normal mouse IgG	Normal mouse IgG		sc-2025		Mouse	200µg/0.5ml	Santa Cruz Biotechnology	Dallas	USA
GAPDH	Glyceraldehyde-3-phosphate dehydrogenase	Mouse, Rat, Human	30202ES80	polyclonal antibody	Rabbit	1:1000	Yeasen Biotechnology	Shanghai	China

Table S2 Reagents information

Reagents	Full name	Density	Citation	Factory
Protein A/G	Protein A/G Magnetic Beads for IP	10 mg/mL	B23202	Selleck
Celastrol	Tripterine	50 mg	S1290	Selleck
MitoSOX™	MitoSOX™ Red mitochondrial superoxide indicator		2544400	Invitrogen
LIVE/DEAD®	LIVE/DEAD® Viability/Cytotoxicity Kit	5 nM	2481050	Invitrogen
Lipo 3000	Lipofectamine™ 3000	/	L3000001	Invitrogen
ATP Assay Kit	Enhanced ATP Assay Kit	4 ml	S0027	Beyotime
JC-1	CBIC2(3)	5 mg/ml	C2005	Beyotime
Tissue Mitochondria Isolation Kit	Tissue Mitochondria Isolation Kit	100/T	C3606	Beyotime
Fluo-8 AM	Fluo-8 AM, green fluorescent calcium binding dye	1 mg	ab142773	Abcam
Rap1 Assay Kit	Rap1 Pull-Down Activation Assay Kit	30/T	81401	NewEast Biosciences
In situ cell death assay kit	Fluorescein-12-UTP tetralithium salt	50/T	11684795910	Roche
Calcein AM	Calcein Red™	5 nM	21900	ATT Bioquest
Cell Mitochondria Isolation Kit	Cell Mitochondria Isolation Kit	50/T	C3601	Beyotime

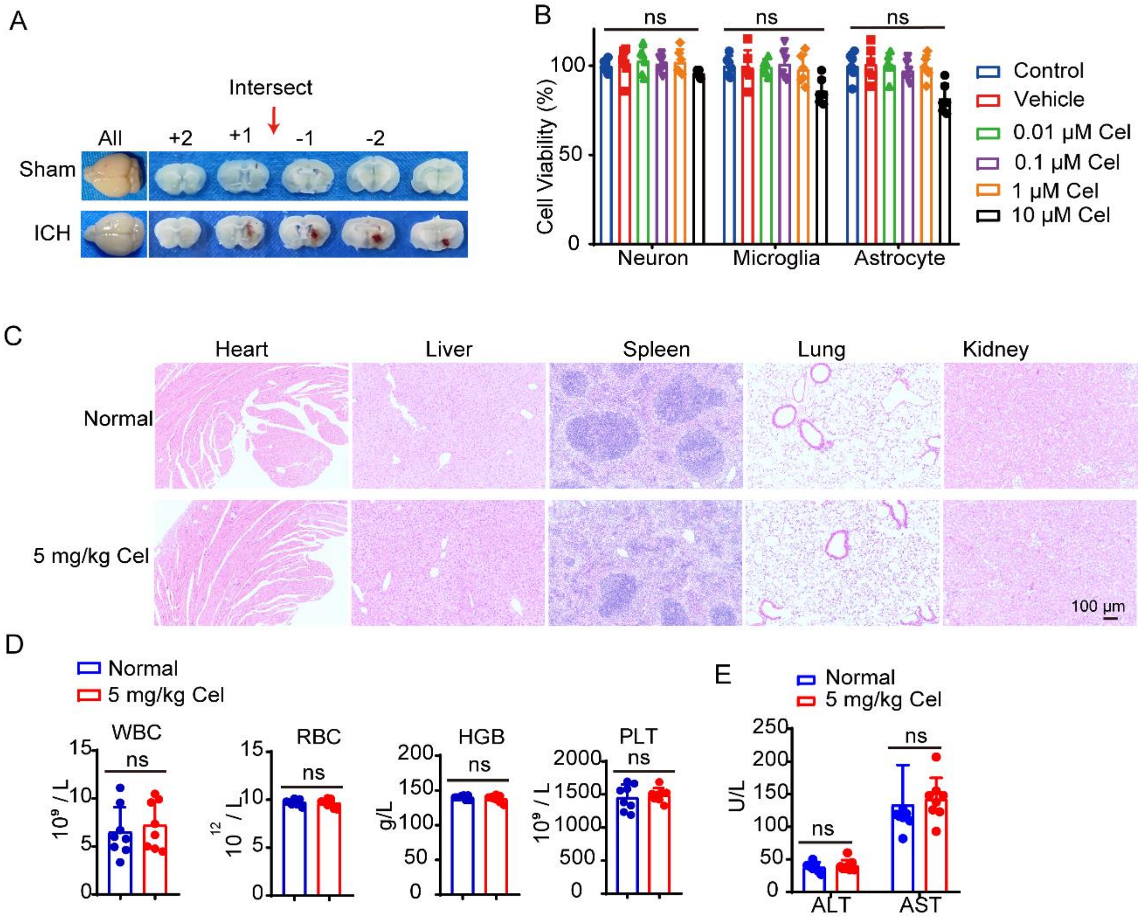

Figure S1 Refinement of the ICH model in mice and evaluation of the side effects and toxicity profile of celastrol. (A) Representative coronal brain sections of mice from both the sham and ICH (24 h) groups are depicted. (B) Neurons, microglia, and astrocytes were subjected to treatment with or without 10 μM celastrol, followed by the MTT assay for assessing cell viability. (C-E) Mice were subjected to intraperitoneal injections of 5 mg/kg celastrol once daily for 3 consecutive days. After a period of 15 days, the heart, liver, spleen, lungs, and kidneys were examined using HE staining to assess the potential toxicity of celastrol (C). (D) Peripheral venous blood was collected from mice for complete blood count analysis, comparing white blood cell (WBC) count, red blood cell (RBC) count, hemoglobin (HGB) levels, and platelet (PLT) count between the two groups. (E)Additionally, serum was isolated to assess levels of alanine transaminase (ALT) and aspartate transaminase (AST).

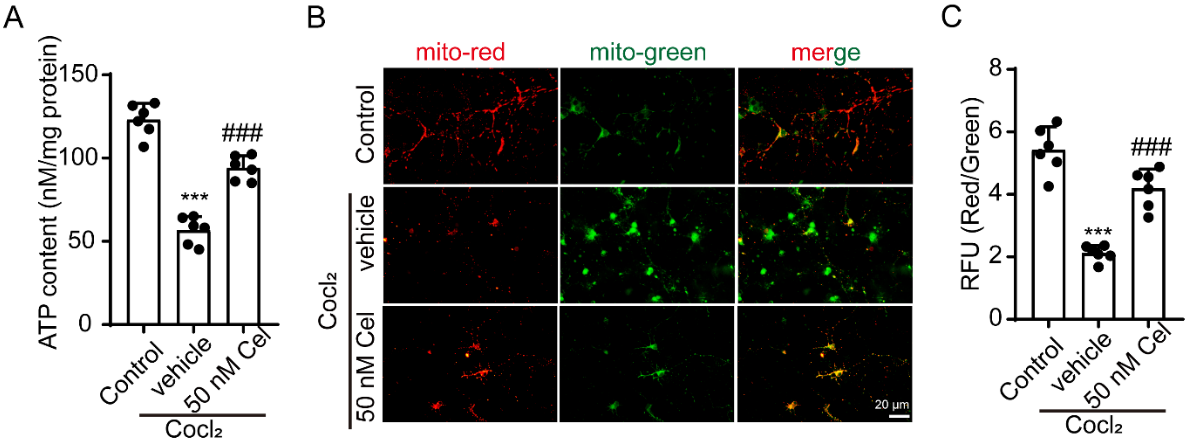

Figure S2 Celastrol improved neuronal mitochondrial dysfunction under hypoxic conditions. Primary neurons were stimulated with 10 μM $Cocl_2$ for 48 h and subsequently exposed to 50 nM celastrol treatment for 24 h. Afterwards, the cells were collected for assessment of mitochondrial function. (A)The ATP content was measured using a chemiluminescence assay, n = 6. (B) Neurons from various experimental groups were subjected to JC-1 staining and subsequently visualized under a Nikon fluorescence microscope. Scale bar: 100 μm. (C) Neurons from different experimental groups were subjected to JC-1 staining, and the fluorescence intensity was quantified using a fluorescent microplate reader with excitation/emission wavelengths of 514/529 nm for monomers and 585/590 nm for aggregates, n = 6. All data are presented as mean ± SD. Statistical significance was determined using one- or two-way ANOVA with Tukey’s multiple comparisons test (***$p < 0.0001$ vs. control group; ###$p < 0.0001$ vs. vehicle group).

One of the consequences of intracerebral hemorrhage (ICH) is the deprivation of brain oxygen, leading to hypoxia. Therefore, we induced a hypoxic condition by treating with cobalt chloride (10 μM for 48 h) and observed that neuronal mitochondria indeed suffered damage under hypoxia. Importantly, our findings demonstrate that celastrol can reverse the decline in MMP and ATP reduction under hypoxic conditions (**Figure S2**).

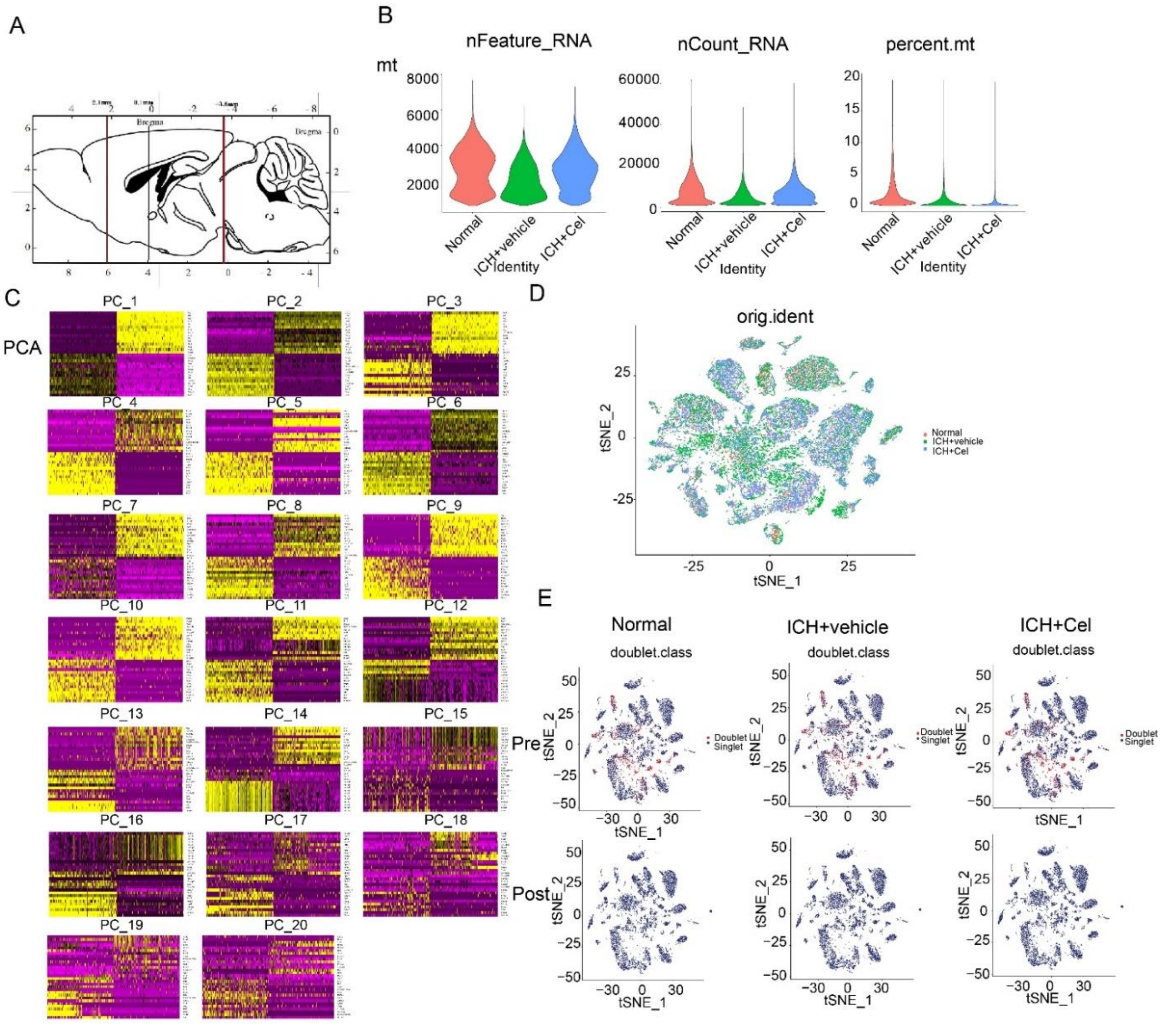

Figure S3 Sampling sites and quality control for single-nucleus RNA sequencing analysis. (A) Sampling locations and strata for murine specimens. (B) The quantification of genes per cell, unique molecular identifier (UMI) counts for individual cells, and the percentage of mitochondrial genes are presented. (C) A PCA plot was generated to visualize the single-cell RNA sequencing data. (D) Each data point represents a single cell, with the color scheme indicating distinct cellular clusters. The column labeled "Orig.ident" denotes the original identity or source of each individual cell sample. (E) A quality control plot comparing the distribution of doublet cells before and after removal is presented.

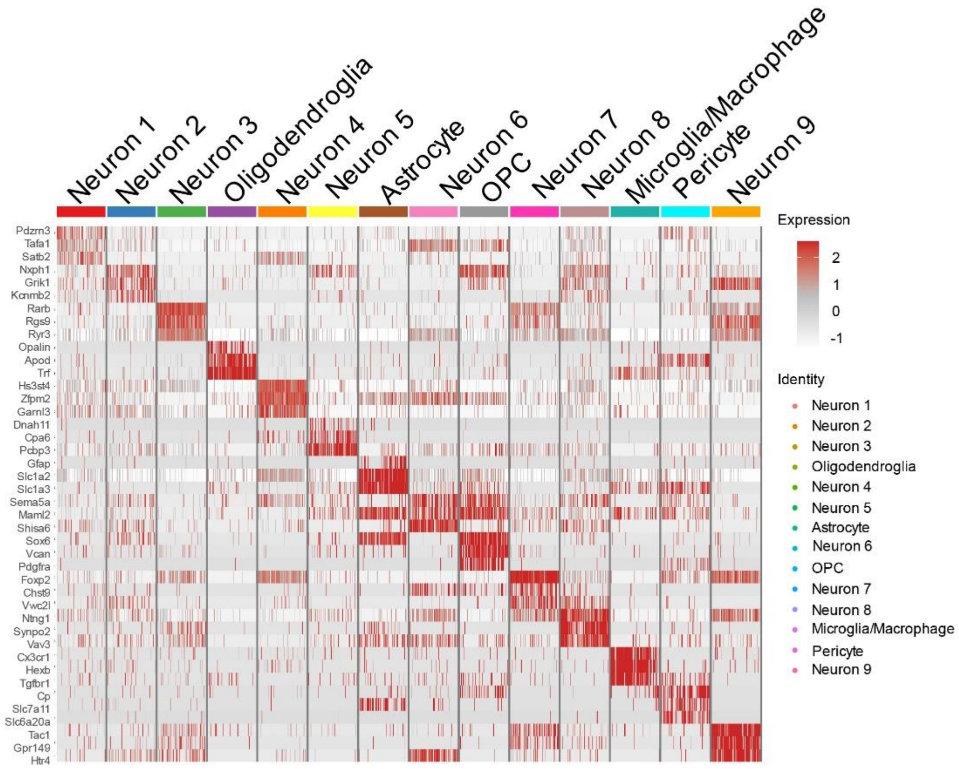

Figure S4 Top genes identified for each specific cell type. The heatmap displays the top 3 normalized expression levels of cell type-specific genes.

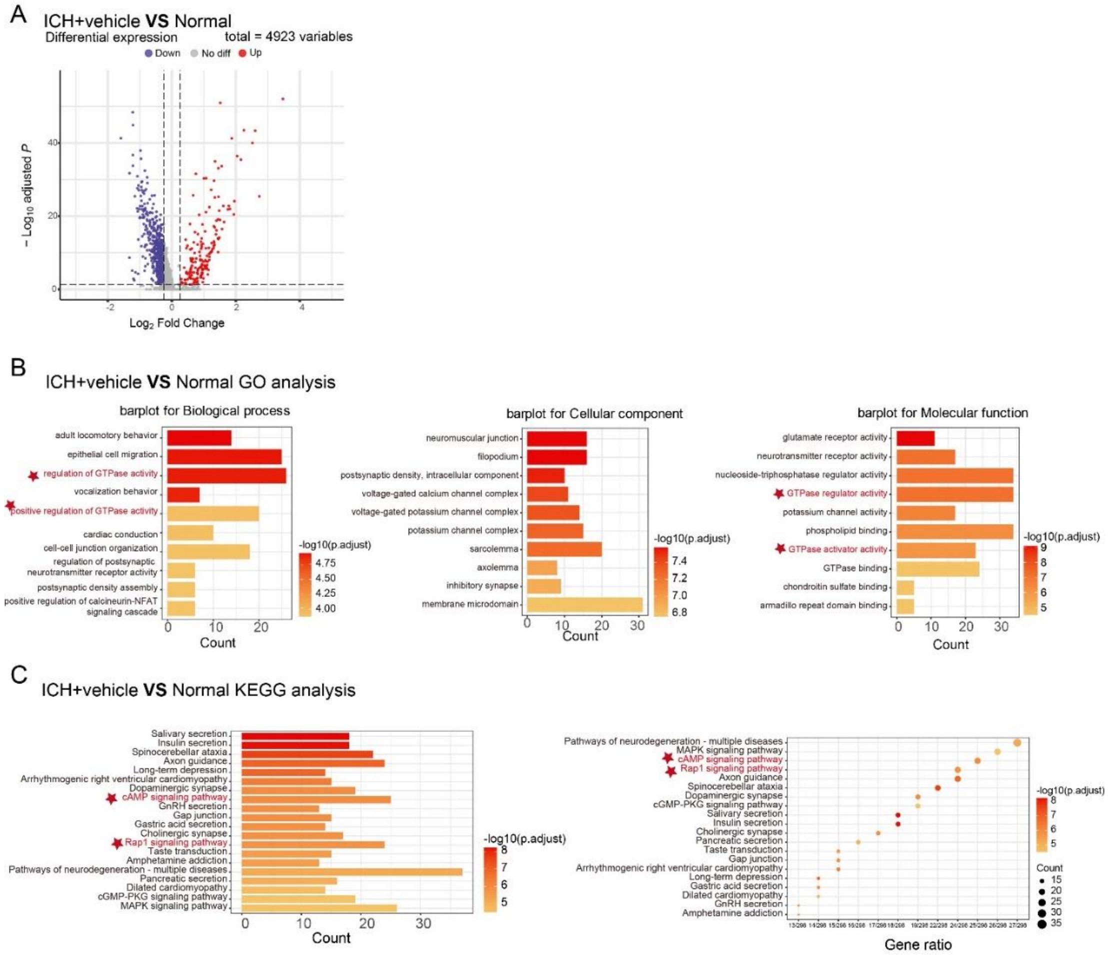

Figure S5 Enrichment analysis between normal and ICH group. (A) The volcano plot illustrates the differential expression of genes between the ICH+vehicle group and the Normal group. (B) An enrichment analysis of GO was conducted to identify potential signaling pathways with an average log2 fold change greater than 0.25 after ICH. (C) KEGG pathway analysis was performed to identify potential signaling pathways following ICH, with an average log2 fold change greater than 0.25.

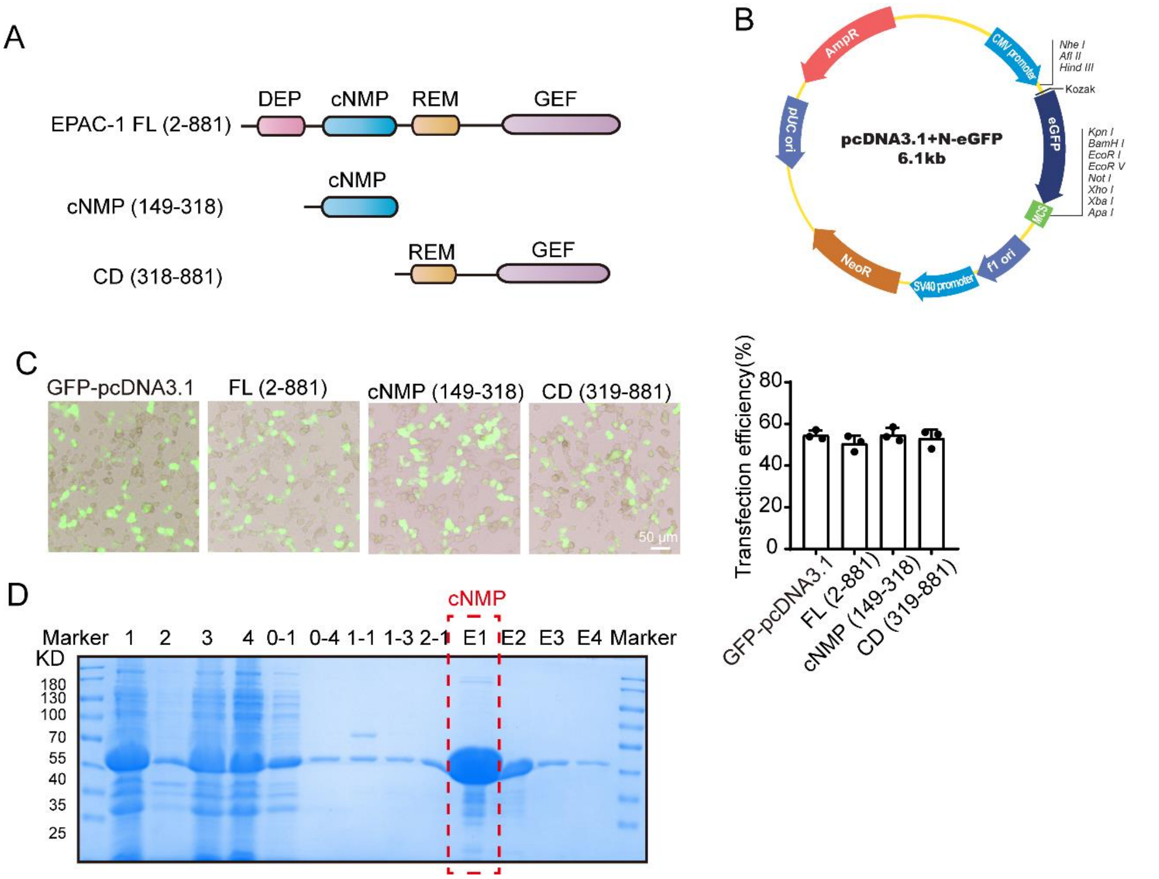

Figure S6 Construction of a truncated EPAC-1 plasmid and purification of cNMP-EPAC1 protein. (A) An illustrative diagram depicting the fragmentation and truncation of EPAC-1 plasmid. (B) Map of the empty vector. (C) 293T cells were transfected with various plasmids for 48 h, and GFP was observed using a Nikon fluorescence microscope. Scale bar = 50 µm. And the transfection efficiency was calculated, n=3 (D) The cNMP-EPAC1 plasmid was constructed in the pGEX-4T vector, where cNMP and pGEX-4T vector GST were fused together, with the GST tag placed at the N-terminal. Then, the prokaryotic expression system was used to express this protein. The lysate, precipitate, flow-through fluid, and lysate supernatant are represented by 1, 2, 3, and 4, respectively. The cleaning process of three different detergents are represented by 0-1, 0-4, 1-1, 1-3, and 2-1, with each tube connected to a volume of 5 mL, while E1, E2, E3, and E4 represent the elution part with each tube connected to a volume of 2 mL.

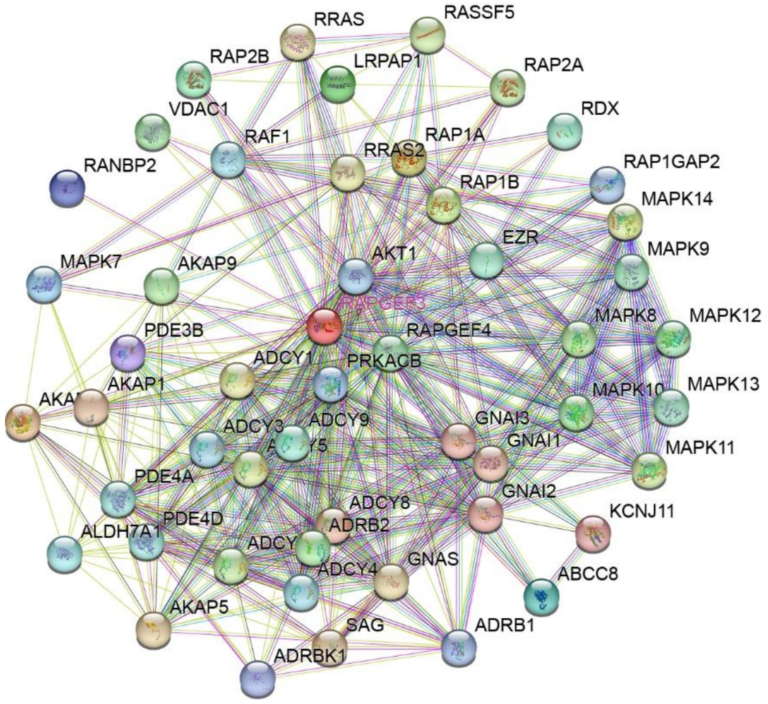

Figure S7 Protein-protein interaction (PPI) for EPAC-1. The PPI network obtained from the STRING database (https://string-db.org) was analyzed to identify potential interacting proteins of EPAC-1.

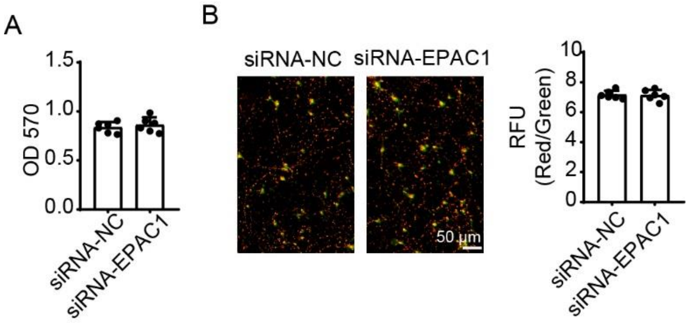

Figure S8 EPAC-1 knockdown did not affect neuronal cell viability or mitochondrial function. Primary neurons were transfected with siRNA-NC or siRNA-EPAC-1. After 48 h, cell viability was assessed using the MTT assay. Neurons from different experimental groups underwent JC-1 staining, and fluorescence intensity was quantified using a fluorescent microplate reader with excitation/emission wavelengths of 514/529 nm for monomers and 585/590 nm for aggregates. All data are presented as mean ± SD. Statistical significance was determined using one-way ANOVA with Tukey's multiple comparisons test (*p < 0.05, **p < 0.001, ***p < 0.0001 vs. control group; #p < 0.05, ##p < 0.001, ###p < 0.0001 vs. vehicle group; n = 6).
